# Novel analytical methods to interpret large sequencing data from small sample sizes

**DOI:** 10.1186/s40246-019-0235-1

**Published:** 2019-08-30

**Authors:** Florence Lichou, Sébastien Orazio, Stéphanie Dulucq, Gabriel Etienne, Michel Longy, Christophe Hubert, Alexis Groppi, Alain Monnereau, François-Xavier Mahon, Béatrice Turcq

**Affiliations:** 1 Laboratory of Mammary and Leukaemic Oncogenesis, Inserm U1218 ACTION, Bergonié Cancer Institute, University of Bordeaux, 146 rue Léo Saignat, bâtiment TP 4ème étage, case 50, 33076 Bordeaux, France; 2 Team EPICENE, Inserm U1219 BPH, Bergonié Cancer Institute, University of Bordeaux, Bordeaux, France; 3 Inserm U1211 MRGM, University of Bordeaux, Bordeaux, France; 4 The Bordeaux Bioinformatics Center (CBiB), University of Bordeaux, Bordeaux, France

**Keywords:** Chronic myeloid leukemia, Next-generation sequencing, Pharmacogenetics, Small sample size, Statistics, Factorial correspondence analysis, Hierarchical clustering on principal components, Rank products

## Abstract

**Background:**

Targeted therapies have greatly improved cancer patient prognosis. For instance, chronic myeloid leukemia is now well treated with imatinib, a tyrosine kinase inhibitor. Around 80% of the patients reach complete remission. However, despite its great efficiency, some patients are resistant to the drug. This heterogeneity in the response might be associated with pharmacokinetic parameters, varying between individuals because of genetic variants. To assess this issue, next-generation sequencing of large panels of genes can be performed from patient samples. However, the common problem in pharmacogenetic studies is the availability of samples, often limited. In the end, large sequencing data are obtained from small sample sizes; therefore, classical statistical analyses cannot be applied to identify interesting targets. To overcome this concern, here, we described original and underused statistical methods to analyze large sequencing data from a restricted number of samples.

**Results:**

To evaluate the relevance of our method, 48 genes involved in pharmacokinetics were sequenced by next-generation sequencing from 24 chronic myeloid leukemia patients, either sensitive or resistant to imatinib treatment. Using a graphical representation, from 708 identified polymorphisms, a reduced list of 115 candidates was obtained. Then, by analyzing each gene and the distribution of variant alleles, several candidates were highlighted such as *UGT1A9*, *PTPN22*, and *ERCC5*. These genes were already associated with the transport, the metabolism, and even the sensitivity to imatinib in previous studies.

**Conclusions:**

These relevant tests are great alternatives to inferential statistics not applicable to next-generation sequencing experiments performed on small sample sizes. These approaches permit to reduce the number of targets and find good candidates for further treatment sensitivity studies.

**Electronic supplementary material:**

The online version of this article (10.1186/s40246-019-0235-1) contains supplementary material, which is available to authorized users.

## Background

Pharmacokinetics refers to drug transport, absorption, or metabolism affecting treatment efficacy. Inter-individual variability in drug response has been described and may be associated with genetic variants (pharmacogenetics) [1]. Identification of these variants, differentially enriched in patients, could help to predict their response to the treatment. However, only a few published studies have reported genetic predictors of efficacy that met the criteria with statistical significance [2, 3]. Targeted approaches were often performed, using single nucleotide polymorphism (SNP) arrays or Sanger sequencing of selected genes. In this way, only common and known variants were investigated. The emergence of next-generation sequencing (NGS) since the 2000s opened up new perspectives and allows to identify rare and non-described variants [4], even though generating highly confident results is still a concern [5, 6]. Small sample sizes are a recurrent issue in pharmacogenetic studies mainly due to a lack of sample availability and high sequencing costs. As the number of sequenced patients is low, conventional statistical approaches are not applicable to highlight the best polymorphisms, differentially expressed or mutated between a control and a treated group. In this study, we propose novel analytical approaches to identify and filter the best variants, more likely to be associated with drug sensitivity, from a massive amount of NGS data using a limited sample size. To assess and to test these approaches, targeted sequencing was performed on chronic myeloid leukemia (CML) patients. CML is a clonal myeloproliferative disorder characterized by the aberrant Philadelphia chromosome, arising from a reciprocal translocation t (9,22) (q34;q11) [7]. This event results in the *BCR-ABL1* (*breakpoint cluster region-Abelson 1*) fusion gene encoding a constitutively active tyrosine kinase that upregulates many kinase pathways. These alterations lead to leukemogenesis: cell proliferation increase, apoptosis inhibition, and persistence of hematopoietic stem cells. This BCR-ABL1 chimeric protein is specifically targeted and inhibited by tyrosine kinase inhibitors (TKIs), such as imatinib mesylate (IM, Gleevec®), commonly used as first-line therapy for CML patients. This treatment shows impressive results with a 10-year event-free survival of 83% in 2013 [8]. Despite these convincing results, resistance to treatment is a persistent clinical issue. Mutations in *ABL1* kinase domain or BCR-ABL1 overexpression are known mechanisms of resistance for about 50% of patients. For other cases, the resistance is still unexplained and surely involves more complex and heterogeneous mechanisms [9]. Notably, alterations of proteins implicated in pharmacokinetics could be participating [10]. To identify genetic variants associated with IM resistance, samples at diagnosis from CML patients, either sensitive or resistant to the IM treatment, were sequenced. Small sample size was available: 12 sensitive patients and 12 resistant patients. Forty-eight genes, selected from previous pharmacogenetic studies, were analyzed by a custom approach using NGS. In this way, all polymorphisms in splicing sites, promoting and coding regions, already described in public databases or new ones, have been identified. They were then filtered and classified according to the variant allele frequency (VAF). Novel approaches using descriptive statistics, simulation studies, and non-parametric statistics were performed to investigate the results generated from this NGS study using a small cohort of patients.

## Results

### Selection of 48 genes involved in pharmacokinetics

Forty-eight genes were selected and sequenced by NGS (Table 1). They encode proteins involved in several pathways potentially linked to IM resistance by directly regulating TKIs or different processes in the leukemic cells. They were classified into six groups. The first group includes 10 genes encoding plasma proteins, membrane transporters, and regulators, involved in the transport and the diffusion of IM through the cell membrane. Genes involved in this process were largely studied in the field of IM resistance. In particular, three exonic polymorphisms (rs1045642 3435C>T, rs1128503 1236C>T, rs2032582 2677G>T/A) in *ATP-binding cassette subfamily B member 1* (*ABCB1* also known as *multidrug resistance protein 1*, *MDR1*), encoding a major IM membrane efflux transporter, have already been identified and associated with lower IM efficiency in several studies, although there are some conflicting results [11, 12]. *ATP-binding cassette subfamily G member 2* (*ABCG2*) and *solute carrier family 22 member 1* (*SLC22A1* also known as *human organic cation transporter type 1*, *hOCT1 or OCT1*), both encoding major IM membrane transporters, are also widely studied, and several non-synonymous polymorphisms in these two genes were associated with a lower IM response [12]. The second group includes 12 genes encoding metabolic enzymes and regulators. IM is mainly metabolized by the cytochrome P450 (CYP) isoenzymes. Polymorphisms in *CYP3A4* and *CYP3A5*, both encoding dominant enzymes involved in IM metabolism, have been described in several studies as correlated with IM resistance in CML patients [12]. The third and fourth groups include genes encoding proteins involved in cell cycle and proliferation regulation (*n* = 5) and proteins involved in DNA repair in response to damages (*n* = 10) notably proteins of the nucleotide excision repair (NER) pathway, associated to treatment efficiency in various diseases. Alterations of these cell processes might impair the IM efficiency by enhancing the capacity of cells to proliferate. The next groups include factors involved in cytokine pathways (*n* = 6) and kinases and phosphatases regulating BCR-ABL1 (*n* = 5); both have been suggested to be involved in IM sensitivity.

**Table 1:** List and characteristics of the 48 sequenced genes (obtained from GeneCards® database)

Gene symbol	Chromosomal location	Gene name
Plasma proteins, membrane transporters, and regulators (*n* = 10)		
ABCB1	7q21.12	ATP-binding cassette subfamily B member 1
ABCC2	10q24	ATP-binding cassette subfamily C member 2
ABCG2	4q22.1	ATP-binding cassette subfamily G member 2 (Junior blood group)
HFE	6p21.3	Hemochromatosis
HIF1A	14q23.2	Hypoxia inducible factor 1 alpha subunit
ORM1	9q32	Orosomucoid 1
SLC22A1	6q25.3	Solute carrier family 22 member 1
SLC22A4	5q23.3	Solute carrier family 22 member 4
SLCO1A2	12p12	Solute carrier organic anion transporter family member 1A2
SLCO1B1	12p12	Solute carrier organic anion transporter family member 1B1
Metabolism enzymes and regulators (*n* = 12)		
CYP1A1	15q24.1	Cytochrome P450 family 1 subfamily A member 1
CYP1A2	15q24.1	Cytochrome P450 family 1 subfamily A member 2
CYP2C19	10q24	Cytochrome P450 family 2 subfamily C member 19
CYP2C8	10q24.1	Cytochrome P450 family 2 subfamily C member 8
CYP2C9	10q24.1	Cytochrome P450 family 2 subfamily C member 9
CYP2D6	22q13.1	Cytochrome P450 family 2 subfamily D member 6
CYP3A4	7q21.1	Cytochrome P450 family 3 subfamily A member 4
CYP3A5	7q21.1	Cytochrome P450 family 3 subfamily A member 5
NR1I2	3q12-q13.3	Nuclear receptor subfamily 1 group I member 2
NR1I3	1q23.3	Nuclear receptor subfamily 1 group I member 3
UGT1A1	2q37.1	UDP glucuronosyltransferase family 1 member A1
UGT1A9	2q37	UDP glucuronosyltransferase family 1 member A9
Cell cycle and proliferation (*n* = 5)		
CCND1	11q13	Cyclin D1
PPP2R2A	8p21.2	Protein phosphatase 2 regulatory subunit *B. alpha*
RPA1	17p13.3	Replication protein A1
RPA2	1p35	Replication protein A2
RPA3	7p21.3	Replication protein A3
DNA repair (*n* = 10)		
ERCC2	19q13.3	Excision repair cross-complementation group 2
ERCC3	2q21	Excision repair cross-complementation group 3
ERCC4	16p13.3	Excision repair cross-complementation group 4
ERCC5	13q22-q34	Excision repair cross-complementation group 5
ERCC6	10q11	Excision repair cross-complementation group 6
ERCC8	5q12.1	Excision repair cross-complementation group 8
LIG1	19q13.33	DNA ligase 1
RAD23B	9p31.2	RAD23 homolog B. nucleotide excision repair protein
XPA	9p22.3	Xeroderma pigmentosum complementation group A
XPC	3p25.1	Xeroderma pigmentosum complementation group C
Cytokine pathways (*n* = 6)		
CXCL8	4q13-q21	C-X-C motif chemokine ligand 8
IFNG	12q14	Interferon gamma
IFNGR1	6q23-q24	Interferon gamma receptor 1
IFNGR2	21q22.1	Interferon gamma receptor 2 (interferon gamma transducer 1)
SOCS1	16p13.13	Suppressor of cytokine signaling 1
SOCS2	12q	Suppressor of cytokine signaling 2
Kinases and phosphatases (*n* = 5)		
AKT1	14q32.33	V-akt murine thymoma viral oncogene homolog 1
ULK3	15q24.1	Unc-51 like kinase 3
PTPN1	20q12.1-q13.2	Protein tyrosine phosphatase non-receptor type 1
PTPN2	18p11.3-p11.2	Protein tyrosine phosphatase non-receptor type 2
PTPN22	1p13.2	Protein tyrosine phosphatase non-receptor type 22

### NGS quality control and genetic polymorphism characteristics

After sequencing of the 48 genes from 24 CML patient samples, around 9 million reads were obtained. Ninety-five percent of the reads passed the quality filter (Phred score over or equal to 20). High read depth was obtained with more than 90% of targeted regions covered with more than 35 short sequences (35X). SNPs and small insertions and deletions (INDELs) were detected and filtered according to the sequencing quality and the depth (30X threshold). After quality filtering, 708 polymorphisms were identified: 41 deletions (the largest of 9 bp), 27 insertions (the largest of 6 bp), and 640 SNPs (Tables 2 and 3, Additional file 1: Table S1).

**Table 2:** ANNOVAR annotations of all sequenced polymorphisms

ANNOVAR annotation	Deletion	Insertion	SNP	Total	Percentage of the total
Upstream to the promoter	0	0	6	6	0.8
Downstream to the promoter	0	0	3	3	0.4
UTR5	1	2	53	56	7.9
UTR3	1	2	29	32	4.5
Exonic	5	1	164	170	24.0
Exonic splicing	0	0	5	5	0.7
Splicing	0	0	1	1	0.2
Intergenic	0	0	3	3	0.4
Intronic	34	22	375	431	60.9
ncRNA_exonic	0	0	1	1	0.2
Total	41	27	640	708	100.0
Percentage of the total	5.8	3.8	90.4	100.0

**Table 3:** ANNOVAR annotations of exonic sequenced polymorphisms

Polymorphisms	Exonic and exonic splicing	Percentage of all exonic polymorphisms	Percentage of all polymorphisms
Deletion	5	2.9	0.7
Frameshift	2	1.1	0.3
Non-frameshift	2	1.1	0.3
Stop-gain	1	0.6	0.1
Insertion	1	0.6	0.1
Frameshift	1	0.6	0.1
SNP	169	96.6	23.9
Non-synonymous	104	59.4	14.7
Synonymous	64	36.6	9.0
Stop-loss	1	0.6	0.1
Total	175	100.0	24.7

### Generating a third group representing non-CML individuals: the general population

The genotypes of the 708 polymorphisms identified in 24 CML patients were reported in a matrix (Additional file 2: Table S2). To highlight the polymorphisms more likely to be involved in IM resistance, these results were compared to allelic frequencies in the general population (non-CML individuals) reported in the 1000 Genomes Project database (1000G, http://www.internationalgenome.org/) [13]. This large sequencing initiative reports the frequency of the alternate allele (AltAF), compared to the human reference genome, for over 88 million variants. This project encompasses the genome of 2504 individuals distributed into 5 sub-populations (phase 3 released in 2014). Patients included in our study were monitored in France. For that reason, only 1000G data from the European sub-population were used for further analyses (EUR, 503 individuals, 201,508 collections, v5b). In the 1000G database, the alternate allele is defined by comparison with the human reference genome. The latter was initially obtained from the whole genome sequencing of one individual and so encompasses major (most frequent in the population) and minor alleles (less frequent in the population) for different polymorphisms. In this way, the alternate allele can be either the major or the minor allele in the population. However, in this study, to highlight variants that may be associated with IM resistance, the minor allele frequency (MAF) needs to be obtained for each polymorphism. Among the 708 identified polymorphisms, 130 polymorphisms did not have the frequency of the alternate allele saved into the 1000G database in the EUR population (Table 4).

**Table 4:** Repartition of the sequenced polymorphisms in 1000G database

	Total polymorphisms	Polymorphisms with AltAF in 1000G	Percentage with AltAF
Upstream to the promoter	6	3	50.0
Downstream to the promoter	3	2	66.7
UTR5	56	52	92.9
UTR3	32	28	87.5
Exonic	170	138	81.2
Exonic splicing	5	3	60.0
Splicing	1	1	100.0
Intergenic	3	3	100.0
Intronic	431	347	80.5
ncRNA_exonic	1	1	100.0
Total	708	578	81.6

**Fig. 1:**
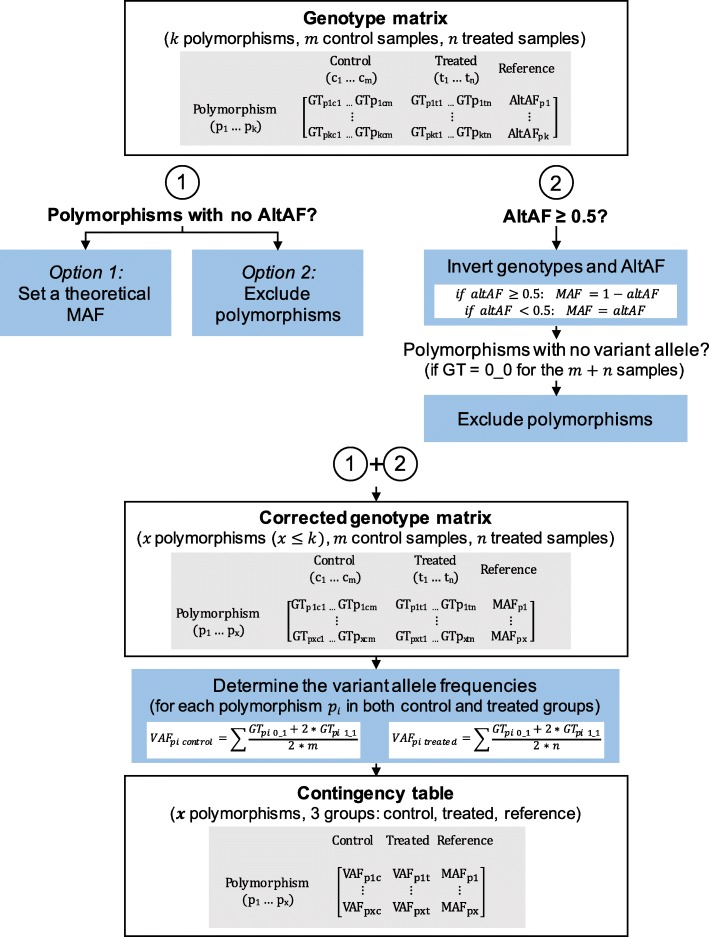
Workflow to adjust the genotype matrix according to the AltAF. First, for polymorphisms with no AltAF, a theoretical MAF is added. Second, genotypes for polymorphisms with an AltAF ≥ 0.5 are inverted. Third, polymorphisms with no variant allele identified by NGS are excluded. Finally, VAFs are determined and reported in a contingency table. GT (genotype) = {0_0, 0_1, 1_1}, 0_0: reference homozygous, 0_1: heterozygous, 1_1: variant homozygous

For the polymorphisms without any AltAF (18.4% of all polymorphisms), an arbitrary MAF was defined. The minor allele was estimated as a rare variant, present in less than one individual in the whole sequenced cohort. As 503 individuals were included in 1000G EUR, the theoretical MAF was 1/503, about 2.10^−3^ [13]. The same analysis could also be performed without setting a theoretical MAF by removing from the dataset the polymorphisms with no AltAF in the 1000G database (data not shown). Furthermore, 439 polymorphisms had an AltAF inferior to 0.5, meaning that the alternate allele was the minor allele in the population. For these polymorphisms, the AltAF was set as the minor allele frequency (MAF). Finally, we identified 139 polymorphisms with an AltAFs equal or superior to 0.5 (24% of all reported AltAFs in 1000G EUR) meaning, in that case, that the alternate allele was the major allele in the reference population. For these polymorphisms, the MAF was defined as 1-AltAF. Moreover, the variant alleles were inverted and the genotypes as well. After this readjustment, 24 polymorphisms showed no variant allele in the 24 CML patients and were removed from the analysis. The VAFs were then determined for both sensitive and resistant CML patients. These frequencies and the MAFs for the 684 identified polymorphisms were reported in a contingency table (Additional file 3: Table S3). All these steps are resumed in Fig. 1.

### Observing individually variant allele distribution using factorial correspondence analysis and hierarchical clustering on principal components

**Fig. 2:**
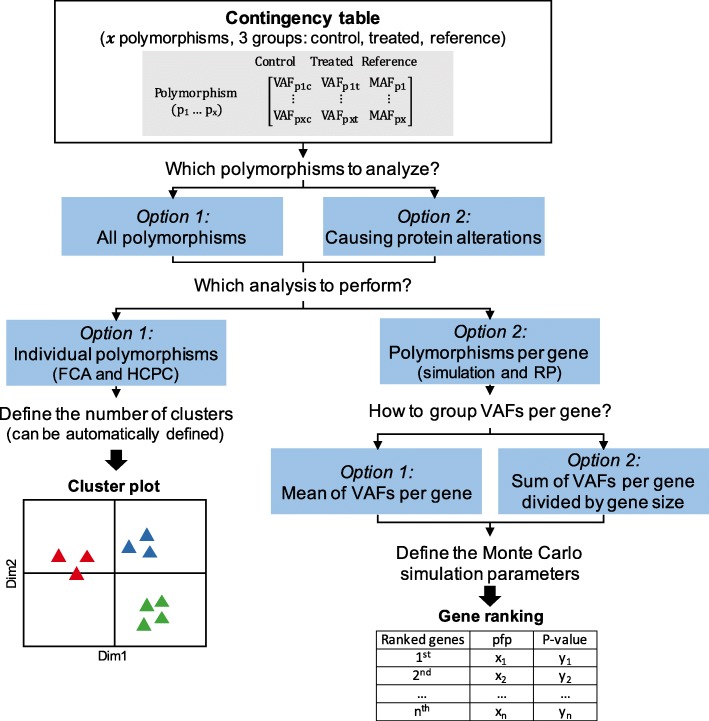
Overview of the two statistical strategies to highlight gene and polymorphisms most likely to be associated with IM resistance. First, either all polymorphisms or only polymorphisms with variant causing protein alterations can be selected. Second, two analyses can be performed. The FCA and HCPC method will display the polymorphisms individually on a two-dimensional graph according to VAFs. The simulation and RP will permit to rank genes according to VAFs (mean of VAFs per gene or sum divided by the size of the gene)

**Fig. 3:**
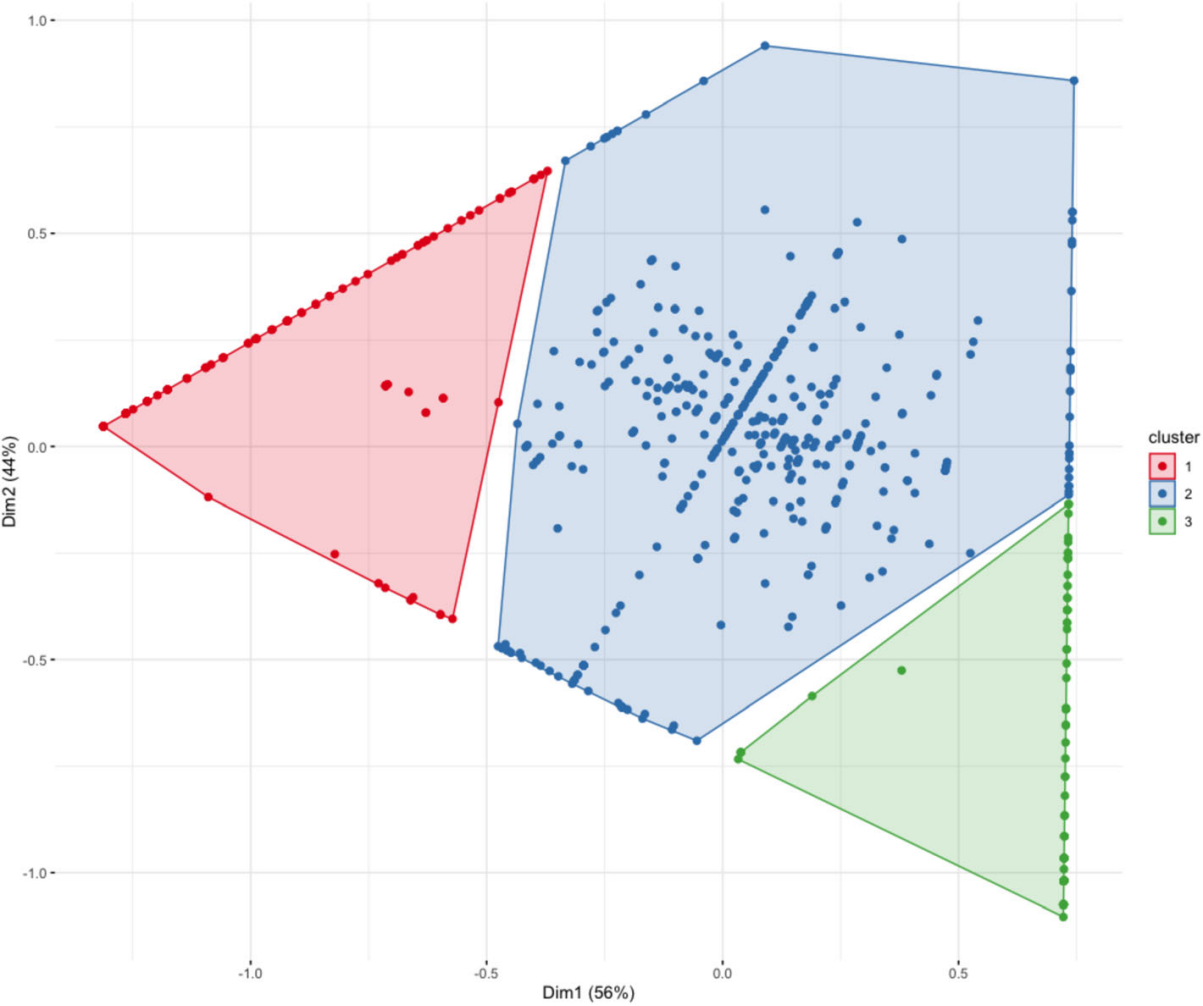
Cluster plot of the distribution of the variant allele for each identified polymorphism (684) among the three groups: CML sensitive patients, CML resistant patients, and the general population. Three clusters were obtained after FCA and HCPC. 1, highest variant frequency in CML sensitive patients; 2, no difference between populations; 3, highest variant frequency in CML resistant patients

Because of the limited number of sequenced patients, the conventional statistical analysis could not be applied. Indeed, in our experiment, common testing assumptions are violated. To overcome this issue, alternative statistical approaches were applied (Fig. 2). First, descriptive statistics were preferentially used and factorial correspondence analysis (FCA) was performed. This approach permitted to display the 684 identified polymorphisms on a two-dimensional graph according to the frequency of the variant allele in each group (sensitive CML patients, resistant CML patients, general population). After hierarchical clustering on principal components (HCPC), three distinct groups were identified (Fig. 3, Additional file 3: Table S3). The cluster 1 contained variants more frequent in CML sensitive patients than in CML resistant patients or in the general population. Otherwise, cluster 3 was comprised of 115 variants enriched in CML resistant patients. These polymorphisms were more likely to be associated with IM resistance because the variant allele was found to be more frequent in these patients than sensitive patients or non-CML patients. For example, a non-synonymous variant (rs2476601, chr1:114377568, 1858G>A, R620W) in the gene encoding the protein tyrosine phosphatase non-receptor type 22 (PTPN22) was highlighted. According to the MAF from 1000G EUR, the variant allele was found in less than 1% in the general population. This variant was enriched in CML resistant patients as 5 out of 12 individuals (21%) were carrying this minor allele whereas all sensitive patients were carrying only the wild-type allele. This polymorphism was also highlighted in 2011 by Guillem et al. and linked to a bad prognosis for CML patients [14]. CML patients with the G/A genotype were seven times more likely to experience a primary failure to IM treatment than the G/G (reference allele) carriers. The FCA and the HCPC displayed globally the variant distribution between the three groups and permitted the discrimination of the different polymorphisms according to the variant allele distribution in CML patients and the general population. Using this approach, from 684 polymorphisms, a reduced list of 115 polymorphisms more likely to be associated with IM resistance was identified.

### Classifying the genes using the rank product method

Secondly, instead of analyzing individual polymorphisms, the variants were grouped together according to the gene they belong to. This approach permitted to identify the genes more likely to be associated with IM resistance because they were carrying multiple genetic variants. The rank product (RP) method was applied (Fig. 2). First, to increase the amount of analyzed data, artificial replicates were generated using a Monte Carlo simulation approach. Then, the genes were ranked according to the frequencies of their variants. Two approaches were tested. Either the sum of frequencies divided by the gene size was performed or the mean of variant frequencies. In both approaches, one variant frequency was obtained for each gene. The results were similar for both calculations (Additional file 4: Table S4): the use of VAF means seems not to generate a bias in the analysis, and it was chosen for the following steps. Several conditions were tested: all polymorphisms, polymorphisms with AltAF in 1000G EUR, or exonic variants causing protein alterations. Results were comparable (Additional file 4: Table S4); however, the last condition was the most biologically relevant. One hundred five polymorphisms in 30 genes were included in the RP test. The three top genes (pfp < 0.05) are reported in Table 5.

**Table 5:** Top genes identified by the rank product method

Gene	RP/Rsum^a^	FC (class 1/class 2)^b^	pfp^c^	*P* value
*UGT1A9*	1.000	0.2495	4.56E−05	1.63E−06
*PTPN22*	2.000	0.4859	4.55E−03	3.25E−04
*ERCC5*	3.000	0.6521	3.45E−02	3.70E−03

^a^RP/Rsum (rank product statistics): the probability that the gene would be classified first in all samples (from both conditions). The lower it is, the more the difference between control and treated conditions is important

^b^FC (class 1/class 2): computed fold change of the average “expression levels” under two conditions

^c^*pfp* percentage of false prediction

The first ranked gene, most significantly variant in CML resistant patients (*P* value< 0.05), was *uridine diphosphate-glucuronosyltransferase family 1 member A9* (*UGT1A9*). The UGT1 enzymes catalyze the transformation of small lipophilic molecules into active metabolites [15]. Interestingly, IM is not a UGT substrate but it can inhibit their activity. Indeed, it has been shown that when IM (same or weaker effect with other TKIs: sorafenib, dasatinib, and nilotinib) was co-administrated with acetaminophen (paracetamol), it decreased its efficacy by inhibiting UGT activity (UGT1A9 and UGT2B15) and so paracetamol glucuronidation [16]. Furthermore, in IM-resistant patients treated by the second-generation TKI, nilotinib, some *UGT1A9* variants affected nilotinib efficacy and were associated with adverse events (hyperbilirubinemia) [17]. This enzyme seems to interact with TKIs, and variants in *UGT1A9* might affect their efficacy and provoke resistance to the treatment. The second gene was, once again, *PTPN22*, already associated with IM resistance in previous studies [14]. The third highlighted gene was *ERCC excision repair 5*, *endonuclease* (*ERCC5*)*.* This gene encodes a factor of the NER pathway, essential to repair DNA lesions such as double-stranded breaks [18]. A non-synonymous variant (rs17655, chr13.103528002, 34829G>C, D1140H) was correlated to a poor response to imatinib in two different studies genotyping 92 and 187 CML patients, respectively [19, 20]. This variant, relatively common in the 1000G EUR population (MAF = 0.25), was found in six resistant patients and only three sensitive patients. As this variant is frequent in the general population, it is less likely to be associated with IM resistance. In our study, it was classified in the cluster number two (no enrichment) in the FCA analysis. Interestingly, the next ranked gene, *XPC* (pfp = 0.1), encodes also a protein of the NER signaling. Guillem et al. described a haplotype (1496C-2815A) correlated to a better prognosis for CML patients [20]. Interestingly, in a cohort composed of 92 patients, the frequency of individuals carrying this haplotype was twofold higher in the sensitive group (61%) than the suboptimal/resistant group (27.5%). In our 24-patient cohort, the rs2228000 variant (1496 T) was found enriched in resistant patients (VAF: resistant = 0.33 vs. sensitive = 0.08) but not the rs2228001 variant (2815C), highly frequent in the three groups (VAF: sensitive = 0.46, resistant = 0.42, general population = 0.40). Using this method, the genes were classified according to their enrichment with non-synonymous variants. A reduced list of three factors more likely to be associated with IM resistance was obtained.

## Discussion

With the NGS emergence, recent pharmacogenetic studies generate massive amounts of data that are difficult to interpret. Despite this, the small sample size is still a recurrent issue. In this way, conventional statistical approaches are not applicable and it is necessary to develop novel analytical approaches to highlight interesting polymorphisms. Here, we proposed several methods to get around inferential statistics limitations using descriptive statistics, simulation method, and non-parametric statistics. These approaches give new paths to follow pharmacokinetic studies. FCA and HCPC are very useful but underused tools to visualize quickly the data and estimate their distribution. In our study, in a simple way, the candidate variant list was reduced from 684 to 115 polymorphisms likely to be associated with IM resistance in CML patients. As it is descriptive statistics, no assumptions are made and there is no statistical limitation in contrary to inferential statistics, today commonly used. The RP method, designed for microarray analysis, seems a powerful tool to classify the candidate genes and again gives clues for further studies. In our study, it was associated with a Monte Carlo simulation to generate simulated experiments and give more weight to the test. It is another way to bypass small sample size limitations. Using this technique, the genes carrying exonic variants causing protein alteration were ranked according to their enrichment in the resistant CML patient group compared to the sensitive CML patient group but also the general population. According to the needs, the list of genes to be ranked can be modulated. In our study, results were comparable while using all the variants or a restricting list of variants more likely to be associated with protein loss of function. Several intriguing genes were highlighted using this method. *UGT1A9* was never directly associated with IM resistance but correlated with other TKI resistance [17]. *PTPN22* was already linked to IM resistance. Notably, a non-synonymous variant (rs2476601, 1858G>A, R620W), previously described, was found enriched in resistant patients in our study [14]. It was included in the cluster enriched in resistant CML patients in the FCA analysis. Finally, *ERCC5* and *XPC* genes involved in NER pathway may also be involved in IM sensitivity.

## Conclusions

In this study, we proposed and tested underused and uncommon statistical strategies. From a large amount of data generated by NGS approaches and few samples, we can highlight interesting targets for future studies. An informatics tool was developed to perform all these analyses in a simple manner and transpose this approach to other NGS experiments performed from small sample sizes.

## Methods

### Patient characteristics

Twenty-four Philadelphia chromosome-positive CML patients newly diagnosed were included in the study either in optimal response (*n* = 12) or in failure response (*n* = 12) according to the European leukemia net (ELN) criteria released in 2013 [21]. None of them carried *BCR-ABL1* alterations at diagnosis. All patients enrolled in this study provided informed consent according to the Declaration of Helsinki. They were daily treated with IM 400 mg. The sex ratio, median age at diagnosis, and Sokal risk group are reported in Table 6.

**Table 6:** Patients’ characteristics

Characteristics	All patients	Optimal response	Failure response
No. of patients (%)	24 (100)	12 (50)	12 (50)
Gender			
Male	15	8	7
Female	9	4	5
Median age at diagnosis (range)	59 (19–86)	61 (19–86)	57 (20–77)
Sokal score (low/intermediate/high)	8/7/9	6/2/4	2/5/5

Overall, there were 9 females (37.5%) and 15 males (62.5%). The median age of patients at diagnosis was 59 years (range 19 to 86 years old). There was no significant difference in ages between males and females or sensitive patients and resistant patients.

### Genomic DNA extraction

Genomic DNA was extracted from leucocyte dry pellets obtained at diagnosis using “DNA extraction kit” (Agilent Technologies) according to the manufacturer’s instructions. The quantity and extraction quality was assessed using a “Nanodrop 2000” spectrophotometer (Thermo Scientific). The genomic DNA integrity (high molecular weight) was verified on a 0.5% agarose gel electrophoresis.

### Targeted sequence capture and next-generation sequencing

The genotyping was performed by NGS. A custom DNA library was prepared using the “SureSelect^QXT^ Target Enrichment for Illumina Multiplexed Sequencing kit” (Agilent technologies). Probes of 120 nucleotides long (Additional file 5: Table S5) were designed using the software “SureDesign” (Agilent Technologies) to specifically capture exons, intron-exon junctions, and promoter regions of the 48 genes of interest (reported in Table 1). A paired-end sequencing (2 × 150 bp) has been performed on a MiSeq device (Illumina) using two “MiSeq Reagent Micro kit v2” (Illumina) according to the manufacturer’s instructions. Obtained sequences were mapped to the human reference assembly GRCh37/hg19 using the Burrows-Wheeler Aligner (BWA) software (http://sourceforge.net/projects/bio-bwa/) [22]. SNPs and small INDELs were identified using the Genome Analysis Toolkit (GATK, http://www.broadinstitute.org/gatk/) [23]. Variant annotation was performed using the Annotation, Visualization, and Impact Analysis (AVIA) online resource (http://avia-abcc.ncifcrf.gov/) [24].

### Contingency table with the observed genotype relative frequencies

To identify polymorphisms correlated with IM resistance, the VAF for each polymorphism was analyzed. Our results were compared to the AltAF depicting the repartition of the variant allele in non-CML individuals. Before applying the different tests, some adjustments were performed as the AltAF shows some limitations. First, some polymorphisms have no AltAF reported; a theoretical MAF was added for these variants. Second, some AltAFs were equal or superior to 0.5; the observed genotypes were inverted for the samples corresponding to these polymorphisms. Third, after these modifications, some polymorphisms had no variant allele in all the samples; these polymorphisms were excluded from the analysis. In the end, a contingency table was obtained. It displayed the VAFs with the polymorphisms reported in rows and the three groups indicated in columns (Fig. 1).

### Factorial correspondence analysis and hierarchical clustering on principal components

An FCA was performed to display the distribution of individuals carrying variant allele in the three groups: sensitive patients, resistant patients, and the general population [25]. This multivariate graphical technique is used to highlight relationships among categorical variables from a contingency table. Unlike common multivariate analyses, this approach makes no distributional assumptions and preserves the categorical nature of the variables. It can be used, without any restriction, to small cohorts. A two-dimensional “map” was obtained with each dot corresponding to one identified polymorphism. An HCPC was then performed [26]. The data were separated into different groups (clusters) according to the closeness of the different points on the graph correlated to the repartition of the variant allele in the three groups. The number of clusters was automatically defined. An unsupervised classification was realized, and clusters were generated. Then, the *k*-means method was applied. The centroid of each cluster was moved according to the average of all the points in the cluster. A new classification was then performed using the new centroid value (Additional file 3: Table S3).

### Monte Carlo simulation and rank product method

The RP method is a non-parametric statistical method, developed to analyze microarray experiments data, to detect genes differentially expressed between two conditions [27, 28]. This approach, based on ranks of fold changes, could also be applied to our experiment. This second method was used to classify the identified genes according to the number and the frequencies of variants found in each one of them. Two approaches were tested. First, the sum of all the VAFs was performed. The number of polymorphisms in one gene can be increased with gene size; therefore, the sum of frequencies was divided by the gene size (values defined in probe design, Additional file 6: Table S6). Second, the mean of all the VAFs for one gene was calculated independently of the gene size. The RP method requires experiment replicates. A Monte Carlo simulation was performed to generate biological replicates from the observed results [29]. A naïve bootstrap was chosen as it is a simulation approach applied in a predicted non-Gaussian subset [30]. Eight simulated experiments were generated (4 from sensitive patients observed data, 4 from resistant patients observed data) with *k* = 999 bootstrap-type resamples for 12 patients each. From these replicates, variant frequencies for each gene (either sum or mean of all the variants per gene) were determined in the two patient groups. For each gene, variant frequencies in CML patient groups were then compared to variant frequencies in the general population by calculating the risk of variant occurrence (ODDS ratio) for each simulated experiments (8 values).

$$ {\mathrm{ODDS}}_i=\frac{\sum_{k=1}^n\frac{q_{ki}\times \left(1-{p}_{ki}\right)}{p_{ki}\times \left(1-{q}_{ki}\right)}}{999} $$

ΣODDS_*i*_ is the plug-in estimation of the ODDS_*i*_ in the CML patient group for *k* = 999 bootstrap resample, *q* is the variant frequency for the gene *i* in the CML patient group, and *p* is the variant frequency for the gene *i* estimated in the general population.

The RP method was applied to these calculated values. First, the ODDS ratios for each gene were compared between sensitive and resistant CML patients (fold changes). Genes were then ranked according to the ODDS ratio differences between sensitive and resistant patient groups (two-class analysis).

### R software

All these analyses were performed using RStudio software (version 1.1.383) and several dedicated packages. A graphic interface was developed to facilitate the use of these novel analytical methods. A detailed manual and the annotated script are given in Additional file 7: File S7.

## Additional files

Additional file 1: **Table S1.** Description of ANNOVAR annotations. (XLSX 13 kb)

Additional file 2: **Table S2.** Original genotype matrix. (CSV 180 kb)

Additional file 3: **Table S3.** Contingency table and list of clusters. (XLSX 36 kb)

Additional file 4: **Table S4.** Rank product results. (XLSX 23 kb)

Additional file 5: **Table S5.** Probe sequences. (XLSX 163 kb)

Additional file 6: **Table S6.** Sizes of the captured genes. (CSV 609 bytes)

Additional file 7: File S7. Detailed manual and annotated script. (DOCX 49 kb)

## Data Availability

Not applicable.

## Abbreviations

1000G: 1000 Genomes Project
ABCB1: ATP-binding cassette subfamily B member 1
ABCG2: ATP-binding cassette subfamily G member 2
AltAF: Alternate allele frequency
BCR-ABL1: Breakpoint cluster region-Abelson 1
CML: Chronic myeloid leukemia
CYP: Cytochrome P450
DNA: Damage recognition and repair factor
ERCC5: ERCC excision repair 5, endonuclease
FCA: Factorial correspondence analysis
HCPC: Hierarchical clustering on principal components
hOCT1: Human organic cation transporter type 1
IM: Imatinib mesylate
INDEL: Insertion and deletion
MAF: Minor allele frequency
MDR1: Multidrug resistance protein 1
NER: Nucleotide excision repair
NGS: Next-generation sequencing
PTPN22: Protein tyrosine phosphatase non-receptor type 22
RP: Rank products
SLC22A1: Solute carrier family 22 member 1
SNP: Single nucleotide polymorphism
TKI: Tyrosine kinase inhibitor
UGT1A9: Uridine diphosphate-glucuronosyltransferase family 1 member A9
VAF: Variant allele frequency
XPC: XPC complex subunit
