# Supplementary material for: Novel analytical methods to interpret large sequencing data from small sample sizes
Source: Hum Genomics. 2019 Aug 30;13:41. doi: 10.1186/s40246-019-0235-1 (PMC6717342; doi:10.1186/s40246-019-0235-1)
Supplement: Supplementary file 7 — File S7. Detailed manual and annotated script. (DOCX 49 kb) [file 40246_2019_235_MOESM7_ESM.docx]

File S7: detailed manual and annotated script

Novel analytical methods to interpret large sequencing data from small sample sizes

**Guide to use the R script + script**

*Before running the script*

Required R packages:

- cluster
- dplyr
- factoextra
- FactoMineR
- graphics
- plyr
- RankProd
- RcmdrMisc
- RecordLinkage
- reshape
- sqldf
- stringr
- tcltk
- tcltk2
- tkrplot

For Mac users, XQuartz must be installed.

This R script is relevant for experiments with the following characteristics:

- Next-generation sequencing (NGS) on diploid human samples
- Two groups of samples (e.g. control and treated)
- Small sample sizes (<30 per group)
- Sequencing of a limited panel of genes

For information, see below the characteristics of our experiment:

- 24 sequenced samples
  - Human patients with chronic myeloid leukemia
  - Comparing 2 groups:
    - 12 samples in the control group (sensitive patients)
    - 12 samples in the treated group (resistant patients)
- 48 captured genes (exon, exon-intron junction, promoter regions)
- Paired-end sequencing (2x150bp) on MiSeq device (Illumina) with two “MiSeq Reagent Micro kit v2” (Illumina)
- Read alignment:
  - Burrows-Wheeler Aligner (BWA) software
  - Mapped to the human reference assembly GRCh37/hg19
- Variant calling:
  - Genome Analysis Toolkit (GATK)
  - SNP and INDELs detected
  - Variant with Phred score >= 20
  - Variant with a depth of coverage >= 30 in at least one sample (allowed because there was a great homogeneity in depth of coverage between samples)
- Variant annotation:
  - Annotation, Visualization and Impact Analysis (AVIA) online resource
  - All annotations databases checked
- Alternate Allele Frequency (AltAF): from the 1000 Genomes project, European sub-population (503 sequenced individuals), August 2015 collection (v5b)

The analyses can be performed from a genotype matrix (.csv file) with the following inputs in this specific order (obtained from the annotated VCF files):

- 1^st^ column: List of the identified polymorphisms (e.g. rs2476601, 1:113834946:G:A)

Note: each row must contain a specific and unique annotation

No required format

- 2^nd^ column: List of the corresponding genes (e.g. *PTPN22*)

Note: for two polymorphisms in the same gene, the gene annotation must be strictly identical

- 3^rd^ column: AltAF for each polymorphism (for missing values either "" or NA)
- 4^th^ to (4+total number of samples)^th^ column: genotype matrix, the observed genotype for each polymorphism and each sample. FIRST control samples, SECOND treated samples.

Note: genotypes must be indicated in the following format

- - - Reference homozygous = 0_0
    - Heterozygous = 0_1
    - Variant homozygous = 1_1
- Last columns: (optional) ANNOVAR annotations for each polymorphism

Example dataset “Table S2. Original genotype matrix.csv”

Sizes of the sequenced genes “Table S6. Sizes of the captured genes.csv”

Other needed information:

- The number of sequenced samples in each group
- The number of individuals sequenced to obtain the AltAF

Note: it depends on the database (e.g. 1000G project, 2504 individuals)

*Description of the script*

The script can be run, all at once on R software version > 3.5.

A series of windows will open successively.

In each window there are some instructions and different options (summary below):

1. Import the genotype matrix (.csv)
2. Indicate the number of samples in each group
3. Polymorphisms with no AltAF. 2 options:
   1. Set a theoretical MAF
   2. Exclude these polymorphisms
4. Automatic calculations.
   1. Invert the genotypes of polymorphisms with an AltAF ≥0.5
   2. Exclude the polymorphisms with no variant allele
5. Choose the polymorphisms to include in the analyses. 2 options:
   1. All polymorphisms
   2. Polymorphisms with variant causing protein alterations
6. Choose the test to perform. 2 options:
   1. FCA and HCPC
   2. Rank products

Several options are proposed for each test.

FCA and HCPC:

1. Number of clusters (automatic: -1)
   1. Generate graphs
   2. Obtain the list of clusters

Rank products:

1. Group the polymorphisms per gene. 2 options:
   1. Mean of variant frequencies per gene
   2. Sum of variant frequencies per gene/gene size (additional file needed)
2. Parameter the rank product test:
   1. Number of samplings (by default: 1000)
   2. Mean of ODDS ratios. Number of replicates per group (by default: 4)
3. Visualize the results
   1. Number of top genes (by default: 10)
   2. Threshold of significance (by default: 0.05 [pfp])
   3. Generate a graph with a threshold of significance (by default: 0.05 [pfp])

*Annotated script*

Every step of the script is explained and may be modified to fit better the experiment (annotations following the #)

| rm(list=ls()) #remove all objects from the current workspace (R memory)  options(max.print=1000000000) #allows management of large dataset  #  #Load packages  library(stringr)  library(reshape)  library(sqldf)  library(RecordLinkage)  library(graphics)  library(RcmdrMisc)  library(dplyr)  library(plyr)  library(RankProd)  library(tcltk)  library(tcltk2)  library(tkrplot)  library(FactoMineR)  library(factoextra)  library(cluster)  #  ##################################  # Window 1 #  # Import the genotype matrix #  ##################################  #  inputs1=function(){  win1=tktoplevel() #Generate a window  tkwm.title(win1,"Import the original genotype matrix") #Define the title of the window    #Define the text font  font=tkfont.create(family="Arial",size=12)  font_b=tkfont.create(family="Arial",size=12,weight="bold")    #Add annotations  label_guideline1=tklabel(win1,text="Rows: polymorphisms identified by NGS",font=font)  label_guideline2=tklabel(win1,text="Columns: samples sequenced by NGS \n",font=font)  label_guideline3=tklabel(win1,text="The matrix reports the observed genotypes for each polymorphism in every sample\n",font=font)  label_guideline4=tklabel(win1,text="The dataset (.csv) must contain the following columns",font=font_b)  label_poly=tklabel(win1,text="1st: polymorphisms",font=font)  label_genes=tklabel(win1,text="2nd: genes",font=font)  label_AltAF=tklabel(win1,text="3rd: AltAF",font=font)  label_samples=tklabel(win1,text="Following columns: sequenced samples and observed genotypes (0_0, 0_1 or 1_1) for each polymorphism",font=font)  label_important=tklabel(win1,text="Report 1st the control samples, 2nd the treated samples\n",font=font_b)    #Function to import the file and save it in R workspace  getcsv=function(){  name=tclvalue(tkgetOpenFile(filetypes="{ {CSV Files} {.csv} } { {All Files} * }"))  data=read.delim(name,stringsAsFactors=FALSE)  assign("data",data,envir=.GlobalEnv)  assign("data_all",data,envir=.GlobalEnv)    label_file=tklabel(win1,text=paste("The file selected is ",name),font=font_b)  label_rows=tklabel(win1,text=paste("The matrix includes ",nrow(data)," polymorphisms (rows)"),font=font)    tkgrid(label_file,sticky="w")  tkgrid(label_rows,sticky="w")  }    #Function to close the window and open the next one  onOK=function(){  tkdestroy(win1)  inputs2()  }    #Create the button objects  import_but=ttkbutton(win1,text="Select CSV file",command=getcsv)  ok_but=ttkbutton(win1,text="Next step",command=onOK)    #Format the window  tkgrid(label_guideline1,sticky="w",row=0,column=0)  tkgrid(label_guideline2,sticky="w",row=1,column=0)  tkgrid(label_guideline3,sticky="w",row=2,columnspan=2)  tkgrid(label_guideline4,sticky="w",row=3,columnspan=2)  tkgrid(label_poly,sticky="w",row=4,column=0)  tkgrid(label_genes,sticky="w",row=5,column=0)  tkgrid(label_AltAF,sticky="w",row=6,column=0)  tkgrid(label_samples,sticky="w",row=7,columnspan=2)  tkgrid(label_important,sticky="w",row=8,columnspan=2)  tkgrid(import_but,sticky="e",row=9,column=0)  tkgrid(ok_but,sticky="w",row=9,column=1)  }  #  ###########################################  # Window 2 #  # Fill the NGS experiment characteristics #  ###########################################  #  inputs2=function(){  win2=tktoplevel()  tkwm.title(win2,"Fill the NGS experiment characteristics")    font=tkfont.create(family="Arial",size=12)  font_b=tkfont.create(family="Arial",size=12,weight="bold")    label_number=tklabel(win2,text="Indicate the number of samples in each group",font=font_b)  label_ncont=tklabel(win2,text="Control group",font=font)  label_ntreat=tklabel(win2,text="Treated group\n",font=font)    #Fill the needed informations  ncontrol=tclVar("")  ntreated=tclVar("")    enter_ncont=tk2entry(win2,textvariable=ncontrol)  enter_ntreat=tk2entry(win2,textvariable=ntreated)    #Function to save the informations in R workspace, close the window and open the next one  submit=function(){  ncontrol=as.numeric(tclvalue(ncontrol))  ntreated=as.numeric(tclvalue(ntreated))  ntotal=ncontrol+ntreated #Calculate the total number of sequenced samples    assign("ncontrol",ncontrol,envir=.GlobalEnv)  assign("ntreated",ntreated,envir=.GlobalEnv)  assign("ntotal",ntotal,envir=.GlobalEnv)    tkdestroy(win2)  inputs3()  }    submit_but=ttkbutton(win2,text="Submit",command=submit)    tkgrid(label_number,sticky="w",row=0,column=0,columnspan=2)  tkgrid(label_ncont,sticky="w",row=1,column=0)  tkgrid(enter_ncont,sticky="w",row=1,column=1)  tkgrid(label_ntreat,sticky="w",row=2,column=0)  tkgrid(enter_ntreat,sticky="w",row=2,column=1)  tkgrid(submit_but,sticky="e",row=3,column=0)  }  #  ##################################################  # Windows 3, 4 and 5 #  # Some polymorphisms have no AltAF #  # Two options: #  # - Set an arbitrary MAF #  # - Remove these polymorphisms from the analysis #  ##################################################  #  inputs3=function(){  win3=tktoplevel()  tkwm.title(win3,"Polymorphisms with no AltAF reported in the database")    font=tkfont.create(family="Arial",size=12)  font_b=tkfont.create(family="Arial",size=12,weight="bold")  font_i=tkfont.create(family="Arial",size=12,slant="italic")    label_one=tklabel(win3,text="Some polymorphisms have no AltAF reported in the preferred database\n",font=font)  label_two=tklabel(win3,text="Two options",font=font_b)  label_option1=tklabel(win3,text="Option 1: Set a theoretical MAF calculated according to the number\n of sequenced individuals in the reference database",font=font)  label_option2=tklabel(win3,text="Option 2: Remove the polymorphisms with no AltAF from the analysis\n",font=font)    #Option 1: set a theoretical MAF to the polymorphisms with no AltAF  option1=function(){  tkdestroy(win3)    win4=tktoplevel()  tkwm.title(win4,"Estimate the theoretical MAF")    label_base=tklabel(win4,text="Indicate the number of individuals sequenced to established the AltAF in the reference database",font=font)  label_base2=tklabel(win4,text="For example, in the 1000 Genomes database, 2504 individuals were included (phase 3)",font=font_i)    #Indicate the number of individuals sequenced to establish the reference database (e.g. 1000 Genomes, 2504 individuals)  nbase=tclVar("")  enter_nbase=tk2entry(win4,textvariable=nbase)    onOK=function(){  nbase=as.numeric(tclvalue(nbase))  assign("nbase",nbase,envir=.GlobalEnv)    theoMAF=1/nbase #The theoretical MAF is 1/total number of sequenced individuals in the general population  data[,3]=replace(data[,3],is.na(data[,3]),theoMAF) #Replace missing AltAF with the theoretical MAF  assign("data",data,envir=.GlobalEnv)  assign("data_theo",data,envir=.GlobalEnv)    tkdestroy(win4)    win5=tktoplevel()  tkwm.title(win5,"")    label_theo=tklabel(win5,text=paste("The theoretical MAF ",theoMAF," has been added for the polymorphisms with no AltAF"),font=font)  label_polyleft=tklabel(win5,text=paste("There are ",nrow(data)," polymorphisms to analyze"),font=font)  onOK2=function(){  tkdestroy(win5)  inputs4()  }    ok_but2=ttkbutton(win5,text="OK",command=onOK2)    tkgrid(label_theo)  tkgrid(label_polyleft)  tkgrid(ok_but2)  }    ok_but=ttkbutton(win4,text="Submit",command=onOK)    tkgrid(label_base)  tkgrid(label_base2)  tkgrid(enter_nbase)  tkgrid(ok_but)  }    #Option 2: remove the polymorphisms with no AltAF from the analysis  option2=function(){  data$AltAFnul=NA #Create an empty column in the dataset  for(i in seq(1,nrow(data))){  if(is.na(data[i,3])){data[i,"AltAFnul"]="nul"}else{data[i,"AltAFnul"]="ok"} #Each time a polymorphism has no AltAF, write "nul" in the "AltAFnul column", otherwise write "ok"  }    data=subset(data,data$AltAFnul=="ok") #Keep only the rows with "ok" therefore the polymorphisms with an AltAF defined in the database  data$AltAFnul=NULL #Remove the column "AltAFnul"    assign("data",data,envir=.GlobalEnv)  assign("data_nul",data,envir=.GlobalEnv)  tkdestroy(win3)    win4=tktoplevel()  tkwm.title(win4,"")    label_win4=tklabel(win4,text="The polymorphisms with no AltAF have been excluded",font=font)  label_win4b=tklabel(win4,text=paste("There are ",nrow(data)," polymorphisms left"),font=font)    onOK=function(){  tkdestroy(win4)  inputs4()  }    ok_but=ttkbutton(win4,text="OK",command=onOK)  tkgrid(label_win4)  tkgrid(label_win4b)  tkgrid(ok_but)  }    opt1_but=ttkbutton(win3,text="Option 1",command=option1)  opt2_but=ttkbutton(win3,text="Option 2",command=option2)    tkgrid(label_one,sticky="w",row=0,column=0)  tkgrid(label_two,sticky="w",row=1,column=0)  tkgrid(label_option1,sticky="w",row=2,column=0)  tkgrid(label_option2,sticky="w",row=3,column=0)    tkgrid(opt1_but,sticky="w",row=4,column=0)  tkgrid(opt2_but,sticky="w",row=4,column=1)  }  #  #####################################################################################  # Windows 6 and 7 #  # Automatic calculations #  # - Some AltAF are equal or superior to 0.5 so genotypes must be inverted #  # - The variant allele frequency for each polymorphism is calculated #  # - The polymorphisms for which no patient is carrying variant are removed #  #####################################################################################  #  #For each polymorphism the reference allele and the variant allele(s) are determined according to an artificial genome  #The human genome used for this analysis is version 37 (hg19)  #Some AltAF are equal or superior to 50% (0.5) and hence correspond to the frequency of the major allele  #In this analysis these polymorphisms are identified and the corresponding genotypes in samples are inverted  #In other words, reference homozygous (0_0) are actually variant homozygous (1_1) carrying two minor alleles instead of two major alleles  #Heterozygous don't change in the matrix  #Variant homozygous (1_1) are actually samples carrying no minor allele, reference homozygous (0_0)  #  inputs4=function(){  win6=tktoplevel()  tkwm.title(win6,"Modify AltAF and calculate variant allele frequency in each group")    font=tkfont.create(family="Arial",size=12)  font_b=tkfont.create(family="Arial",size=12,weight="bold")  font_i=tkfont.create(family="Arial",size=12,slant="italic")    label1=tklabel(win6,text="Modify AltAF",font=font_b)  label2=tklabel(win6,text="Some AltAF are equal or superior to 0.5 and hence correspond to the major allele",font=font)  label3=tklabel(win6,text="These polymorphisms are detected and the different observed genotypes are inverted",font=font)  label4=tklabel(win6,text="(Variant allele are actually major allele and vice versa)",font=font)  label5=tklabel(win6,text="Following this step, some polymorphisms with no minor allele in all the samples are removed\n",font=font_i)  label6=tklabel(win6,text="Calculate the variant allele frequency for each polymorphism",font=font_b)  label7=tklabel(win6,text="These calculations are performed for the three groups (control, treated, general population)\n",font=font)    onOK=function(){  tkdestroy(win6)  i=1  j=4    #Screen the whole matrix to identify AltAF >=0.5 and invert the genotypes to the corresponding polymorphism  for(i in seq(1,nrow(data))){  if(data[i,3]>=0.5){ #Find the rows (polymorphisms) with an AltAF >=0.5  for(j in seq(4,ntotal+3)){  if(data[i,j]=="0_0"){data[i,j]=2}} #Replace the genotype 0_0 with 2 (2 minor alleles) for each concerned sample  for(j in seq(4,ntotal+3)){  if(data[i,j]=="1_1"){data[i,j]=0}} #Replace the genotype 1_1 with 0 for each concerned sample  }    #In a similar manner screen the whole matrix to identify AltAF <0.5 and replace 0_0 by 0 and 1_1 by 2  if(data[i,3]<0.5){  for(j in seq(4,ntotal+3)){  if(data[i,j]=="0_0"){data[i,j]=0}}  for(j in seq(4,ntotal+3)){  if(data[i,j]=="1_1"){data[i,j]=2}}  }}  rm(i)  rm(j)    i=1  j=4    #Replace all the heterozygous genotypes (0_1) by 1  for(i in seq(1,nrow(data))){  for(j in seq(4,ntotal+3)){  if(data[i,j]=="0_1"){data[i,j]=1}  }}  rm(i)  rm(j)    #Generate an artificial MAF: if AltAF>=0.5, MAF = 1-AltMAF  data[,3]=as.numeric(data[,3]) #Set the AltAF as numeric variables  data$AltAF=ifelse(data[,3]>=0.5,(1-data[,3]),data[,3]) #Identify the polymorphism with an AltAF>=0.5 and inverse the MAF    #Calculate the variant allele frequency for each polymorphism in the control and the treated groups  #This step is not necessary for the rank product analysis but still, it is helpful for the rest of the script  data$control_q=NA #Empty column for the frequencies in the control group  data$treated_q=NA #Empty column for the frequencies in the treated group    #Screen the whole matrix and calculate the sum of variant alleles for each polymorphism. Save the data in the control_q column  i=1  j=4  for(i in seq(1,nrow(data))){  num=0  end=ncontrol+3  for(j in seq(4,end)){ #Columns corresponding to the control samples  if(data[i,j]==1){ #Identify the heterozygous individuals  num=num+1 #Add 1 to the sum variable for the next sample with a variant allele  }  if(data[i,j]==2){ #Identify the variant homozygous individuals  num=num+2 #Add 2 to the sum variable for the next sample with a variant allele  }  data[i,"control_q"]=num #Add the result to the sum column  }}  rm(i)  rm(j)  rm(num)    #Similar calculations for the treated samples  i=1  j=4+ncontrol  for(i in seq(1,nrow(data))){  num=0  start=4+ncontrol  end=ncontrol+ntreated+3  for(j in seq(start,end)){ #Columns corresponding to the treated samples  if(data[i,j]==1){  num=num+1  }  if(data[i,j]==2){  num=num+2  }  data[i,"treated_q"]=num  }}  rm(i)  rm(j)  rm(num)    #As some genotypes were inverted, it is possible that, for some polymorphisms, no sample is carrying the minor allele  #These polymorphisms must be removed from the analysis  data$zero=NA #Empty column to tag the row with samples with no minor allele in both control and treated groups    #Screen the whole matrix to identify these polymorphisms  i=1  for(i in seq(1,nrow(data))){  if((data[i,"control_q"]==0)&& (data[i,"treated_q"]==0)){  data[i,"zero"]=0}else{data[i,"zero"]="ok"} #Indicate 0 in the "zero" column if no samples is carrying the minor allele  }  rm(i)    data=subset(data,data$zero=="ok") #Keep only the rows with "ok" therefore at least one sample is carrying minor allele for the corresponding polymorphism  data$zero=NULL #Remove the "zero" column  data=replace(data,is.na(data),0) #Replace NA value by 0    #The variant allele frequencies for each polymorphism in both control and treated groups are calculated  data$control_q=data$control_q/(ncontrol*2)  data$treated_q=data$treated_q/(ntreated*2)    #When all these steps are completed, the matrix is ready for the following statistical tests  #The two tests can be completed independently  assign("data",data,envir=.GlobalEnv)  assign("data_correct",data,envir=.GlobalEnv)    win7=tktoplevel()  tkwm.title(win7,"")    label_left=tklabel(win7,text=paste("There are ",nrow(data)," polymorphisms remaining"),font=font)    onOK2=function(){  tkdestroy(win7)  inputs5()  }    ok2_but=ttkbutton(win7,text="OK",command=onOK2)    tkgrid(label_left)  tkgrid(ok2_but)    }    ok_but=ttkbutton(win6,text="OK",command=onOK)    tkgrid(label1,sticky="w")  tkgrid(label2,sticky="w")  tkgrid(label3,sticky="w")  tkgrid(label4,sticky="w")  tkgrid(label5,sticky="w")  tkgrid(label6,sticky="w")  tkgrid(label7,sticky="w")  tkgrid(ok_but)  }  #  #####################################################  # Windows 8, 9 and 10 #  # Choose the polymorphisms included in the analyses #  # - All polymorphisms #  # - With variant causing protein alterations #  #####################################################  #  inputs5=function(){  win8=tktoplevel()  tkwm.title(win8,"Choose the polymorphisms to analyze")    font=tkfont.create(family="Arial",size=12)  font_b=tkfont.create(family="Arial",size=12,weight="bold")    label_options=tklabel(win8,text="Two options",font=font_b)  label_opt1=tklabel(win8,text="Option 1: analyze all the polymorphisms",font=font)  label_opt2=tklabel(win8,text="Option 2: analyze the polymorphisms with variants causing protein alterations:\nexonic non synonymous, frameshift, stopgain, stoploss\n",font=font)    #Option 1: all polymorphisms included  option1=function(){  assign("data",data,envir=.GlobalEnv)  assign("data_keepall",data,envir=.GlobalEnv)  tkdestroy(win8)    win9=tktoplevel()  tkwm.title(win9,"Option 1: all polymorphisms")    label_ok=tklabel(win9,text=paste("There are ",nrow(data)," polymorphisms"),font=font)    onOK=function(){  tkdestroy(win9)  inputs6()  }    ok_but=ttkbutton(win9,text="OK",command=onOK)    tkgrid(label_ok,sticky="w",row=0,column=0)  tkgrid(ok_but)  }    #Option 2 : polymorphisms with variants causing protein alterations  option2=function(){  tkdestroy(win8)    win9=tktoplevel()  tkwm.title(win9,"Option 2: variants causing protein alterations")    label_annot=tklabel(win9,text="Indicate the exact name of the column containing the ANNOVAR annotations",font=font)    name_annot=tclVar("")  enter_name=tk2entry(win9,textvariable=name_annot)    onOK=function(){  name_annot=tclvalue(name_annot)  assign("name_annot",name_annot,envir=.GlobalEnv)  tkdestroy(win9)    #Remove from the dataset, polymorphisms with polymorphisms not causing protein alterations (according to ANNOVAR annotation)  data$isalteration=NA #Create an empty column in the dataset to indicate if the polymorphism is causing protein alterations  alteration=c("nonsynonymous","frameshift","stopgain","stoploss") #Create a vector containing the word to look for in the annotation column    #Screen the annotation column to identify the variants causing protein alterations  i=1  j=1  for(i in seq(1,nrow(data))){  for(j in 1:4){  if(str_detect(data[i,name_annot],alteration[j])){  data[i,"isalteration"]="ok" #Write "ok" if it is, if not "NA"  }}}    data=subset(data,data$isalteration=="ok") #Keep only the rows with "ok"  data$isalteration=NULL  rm(i)  rm(j)    #Some frameshift can remain. They are removed according to the same principle  data$isNF=NA    i=1  for(i in seq(1,nrow(data))){  if(str_detect(data[i,name_annot],"nonframeshift")){  data[i,"isNF"]="ok"  }}  rm(i)    data=subset(data,is.na(data$isNF)) #Remove the "non frameshift" variants  data$isNF=NULL    win10=tktoplevel()  tkwm.title(win10,"")    label_ok=tklabel(win10,text=paste("There are ",nrow(data)," remaining polymorphisms"),font=font)    onOK=function(){  assign("data",data,envir=.GlobalEnv)  assign("data_alter",data,envir=.GlobalEnv)  tkdestroy(win10)  inputs6()  }    ok_but=ttkbutton(win10,text="OK",command=onOK)    tkgrid(label_ok)  tkgrid(ok_but)  }    ok_but=ttkbutton(win9,text="OK",command=onOK)    tkgrid(label_annot)  tkgrid(enter_name)  tkgrid(ok_but)  }    opt1_but=ttkbutton(win8,text="Option 1",command=option1)  opt2_but=ttkbutton(win8,text="Option 2",command=option2)    tkgrid(label_options,sticky="w",row=0,column=0)  tkgrid(label_opt1,sticky="w",row=1,column=0)  tkgrid(label_opt2,sticky="w",row=2,column=0)  tkgrid(opt1_but,sticky="w",row=3,column=0)  tkgrid(opt2_but,sticky="w",row=3,column=1)  }  #  ########################################################################################  # Window 11 #  # Choose the statistical analysis to perform #  # Two options: #  # - FCA and HCPC: visualize the distribution of polymorphisms between the three groups #  # according to the frequency of variant allele #  # - Rank products: rank the genes according to the frequency of variant alleles of #  # all the polymorphisms present in the corresponding genes #  ########################################################################################  #  inputs6=function(){  win11=tktoplevel()  tkwm.title(win11,"Choose the statistical analysis to perform")    font=tkfont.create(family="Arial",size=12)  font_b=tkfont.create(family="Arial",size=12,weight="bold")    label_two=tklabel(win11,text="Two analyses can be performed on the dataset",font=font)  label_FCA1=tklabel(win11,text="\nFactorial correspondence analysis and hierarchical clustering on principal components",font=font_b)  label_FCA2=tklabel(win11,text="It allows to visualize the repartition of the polymorphisms in the 3 groups according to the variant allele frequency",font=font)  label_RP1=tklabel(win11,text="\nRank products",font=font_b)  label_RP2=tklabel(win11,text="It allows to rank the genes according to the frequency of variant alleles in the corresponding gene",font=font)  label_RP3=tklabel(win11,text="Resistant patients are carrying more variants in the top ranked genes",font=font)    FCA=function(){  tkdestroy(win11)  inputs7()  }    RP=function(){  tkdestroy(win11)  inputs8()  }    FCA_but=ttkbutton(win11,text="FCA and HCPC",command=FCA)  RP_but=ttkbutton(win11,text="Rank products",command=RP)    tkgrid(label_two,sticky="w",row=0,column=0)  tkgrid(label_FCA1,sticky="w",row=2,column=0)  tkgrid(label_FCA2,sticky="w",row=3,column=0)  tkgrid(FCA_but,row=4,column=0)  tkgrid(label_RP1,sticky="w",row=5,column=0)  tkgrid(label_RP2,sticky="w",row=6,column=0)  tkgrid(label_RP3,sticky="w",row=7,column=0)  tkgrid(RP_but,row=8,column=0)  }  #  ###########################################################  # Windows 12, 13 and 14 #  # Factorial correspondence analysis (FCA) and #  # Hierarchical clustering on principal components (HCPC) #  ###########################################################  #  inputs7=function(){  #Create a table with 4 columns:  #The list of each sequenced polymorphisms and the gene associated  #The variant allele frequencies in each group (control, treated, general population)    poly=paste(data[,1],data[,2],sep="_") #Create the first column with the polymorphism and the gene  control=data$control_q #Variant allele frequencies of control samples  treated=data$treated_q #Variant allele frequencies of treated samples  general=data$AltAF #Variant allele frequencies of individuals from the general population  data_FCA=data.frame(poly,control,treated,general,stringsAsFactors=FALSE) #New matrix for the FCA analysis  row.names(data_FCA)=data_FCA[,1] #Name of rows = name of polymorphisms+gene  data_FCA=data_FCA[,-1] #Remove the first column with the polymorphisms+gene  assign("data_FCA",data_FCA,envir=.GlobalEnv)    #Perform the factorial correspondence analysis and the clustering  win12=tktoplevel()  tkwm.title(win12,"FCA and HCPC")    font=tkfont.create(family="Arial",size=12)  font_b=tkfont.create(family="Arial",size=12,weight="bold")    label_cluster=tklabel(win12,text="Define the number of expected clusters",font=font_b)  label_default=tklabel(win12,text="Enter -1 to generate the suggested number of clusters",font=font)  label_dataclust=tklabel(win12,text="\nExtract the list of polymorphisms and the corresponding cluster number",font=font)    ncluster=tclVar("")  enter_ncluster=tk2entry(win12,textvariable=ncluster)    graph=function(){  ncluster=as.numeric(tclvalue(ncluster))  assign("ncluster",ncluster,envir=.GlobalEnv)    CA=CA(data_FCA,graph=FALSE) #Factorial correspondence analysis  HCPC=HCPC(CA,nb.clust=ncluster,graph=FALSE) #Hierarchical clustering on principal components  clust=fviz_cluster(HCPC,geom="point", repel=TRUE, show.clust.cent=FALSE, shape=20, pointsize=3,palette="Set1",ggtheme=theme_minimal(),main=NULL) #Generate the graph  assign("FCA",CA,envir=.GlobalEnv)  assign("HCPC",HCPC,envir=.GlobalEnv)  assign("clust",clust,envir=.GlobalEnv)    win13=tktoplevel()  tkwm.title(win13,"The graph has been generated")    label_info=tklabel(win13,text="To visualize the graph, enter 'clust' on the R workspace",font=font)  label_info2=tklabel(win13,text="If needed, you can change the number of clusters and generate another graph",font=font)    ok_but=ttkbutton(win13,text="OK",command=function()tkdestroy(win13))    tkgrid(label_info)  tkgrid(label_info2)  tkgrid(ok_but)  }    dataclust=function(){  dataclust=HCPC$data.clust  assign("dataclust",dataclust,envir=.GlobalEnv)    win14=tktoplevel()  tkwm.title(win14,"")    label_win14=tklabel(win14,text="To visualize the dataset, enter 'dataclust' on the R workspace",font=font)    ok_but=ttkbutton(win14,text="OK",command=function()tkdestroy(win14))    tkgrid(label_win14)  tkgrid(ok_but)  }    RP_but=function(){  tkdestroy(win12)  inputs8()  }    graph_but=ttkbutton(win12,text="Generate the FCA graph",command=graph)  dataclust_but=ttkbutton(win12,text="Data polymorphisms with clusters",command=dataclust)  RP_but=ttkbutton(win12,text="Rank products",command=RP_but)  end_but=ttkbutton(win12,text="Close",command=function()tkdestroy(win12))      tkgrid(label_cluster,sticky="w",row=0,column=0)  tkgrid(label_default,sticky="w",row=1,column=0)  tkgrid(enter_ncluster,sticky="w",row=2,column=0)  tkgrid(graph_but,sticky="w",row=3,column=0)  tkgrid(label_dataclust,sticky="w",row=4,column=0)  tkgrid(dataclust_but,sticky="w",row=5,column=0)  tkgrid(RP_but,sticky="w",row=6,column=0)  tkgrid(end_but,sticky="w",row=6,column=1)  }  #  ##########################  # Rank product analysis #  # Several steps #  ##########################  #  ###########################################################  # Windows 15, 16 and 17 #  # Generate a table with variant frequencies #  # in the general population for each gene #  # Two options to rank the genes #  # - Mean of variant allele frequencies per gene #  # - Sum of variant allele frequencies per gene/size of the gene #  ###########################################################  #  inputs8=function(){  win15=tktoplevel()  tkwm.title(win15,"Rank product analysis: choose the method to group the polymorphisms and rank the genes")    font=tkfont.create(family="Arial",size=12)  font_b=tkfont.create(family="Arial",size=12,weight="bold")  font_i=tkfont.create(family="Arial",size=12,slant="italic")    label_options=tklabel(win15,text="Two options",font=font_b)  label_opt1=tklabel(win15,text="Option 1: calculate the mean of variant allele frequencies of all the variants in one gene",font=font)  label_opt2=tklabel(win15,text="Option 2: calculate the sum of variant allele frequencies of all the variants divided by the gene size\n",font=font)    #Generate a dataset with the list of the genes and the mean/sum of variant frequencies in the general population  data_pop=data  data_pop[,2]=gsub(",","",data_pop[,2])  colnames(data_pop)[2]="gene"  data_pop=ddply(data_pop,.(gene),mutate,  p=sum(AltAF)) #Sum of variant frequencies per gene  nb_poly=data.frame(table(data_pop$gene),stringsAsFactors=FALSE) #Count the number of polymorphisms per gene and the total number of genes  colnames(nb_poly)=c("gene","nb_poly")  data_pop$dup=duplicated(data_pop$gene) #Identify the first line for each gene  data_pop=subset(data_pop,data_pop$dup=="FALSE") #Keep only the first line for each gene  data_pop$dup=NULL    #Mean of the variant frequencies per gene  option1=function(){  #Divide the frequency per gene with the number of polymorphisms per gene  data_pop=merge(data_pop,nb_poly,by="gene") #Add the column with the number of polymorphisms per gene to the dataset  data_pop$p=data_pop$p/data_pop$nb_poly #Divide variant allele frequency (per gene) with the number of polymorphisms (per gene)  data_pop=cbind(data_pop$gene,data_pop$p)  data_pop=data.frame(data_pop,stringsAsFactors=FALSE)  colnames(data_pop)=c("gene","p")  data_pop[,1]=as.character(data_pop[,1])  data_pop[,2]=as.numeric(data_pop[,2])    assign("data_pop",data_pop,envir=.GlobalEnv)  assign("data_pop_mean",data_pop,envir=.GlobalEnv)  tkdestroy(win15)    win16=tktoplevel()  tkwm.title(win16,"")    label_ok1=tklabel(win16,text=paste("There are ",nrow(data_pop)," genes included in the analysis"),font=font)    onOK=function(){  option="mean"  assign("option",option,envir=.GlobalEnv)  tkdestroy(win16)  inputs9()  }    ok_but=ttkbutton(win16,text="OK",command=onOK)    tkgrid(label_ok1)  tkgrid(ok_but)  }    #Sum of the variant frequencies per gene / size of the gene  option2=function(){  tkdestroy(win15)  win16=tktoplevel()  tkwm.title(win16,"Import a dataset detailing sizes of each gene")    label_import=tklabel(win16,text="Import a table with two columns",font=font_b)  label1=tklabel(win16,text="1st: gene names (the same than the matrix)",font=font)  label2=tklabel(win16,text="2nd: size of the genes\n",font=font)    getcsv=function(){  name=tclvalue(tkgetOpenFile(filetypes="{ {CSV Files} {.csv} } { {All Files} * }"))  size=read.csv2(name,stringsAsFactors=FALSE)  assign("size",size,envir=.GlobalEnv)    label_file=tklabel(win16,text=paste("The file selected is ",name),font=font_b)  label_rows=tklabel(win16,text=paste("The list contains ",nrow(size)," genes (rows)"),font=font)    tkgrid(label_file,sticky="w")  tkgrid(label_rows,sticky="w")  }    onOK=function(){  tkdestroy(win16)  colnames(size)=c("gene","size")  data_pop=merge(data_pop,size,by="gene")    #Divide the sum of frequencies per gene with the size of the gene  data_pop$p=data_pop$p/data_pop$size  data_pop=cbind(data_pop$gene,data_pop$p)  colnames(data_pop)=c("gene","p")  data_pop=data.frame(data_pop,stringsAsFactors=FALSE)  data_pop[,1]=as.character(data_pop[,1])  data_pop[,2]=as.numeric(data_pop[,2])    assign("data_pop",data_pop,envir=.GlobalEnv)  assign("data_pop_sum",data_pop,envir=.GlobalEnv)  tkdestroy(win16)    win17=tktoplevel()  tkwm.title(win17,"")    label_ok1=tklabel(win17,text=paste("There are ",nrow(data_pop)," genes included in the analysis"),font=font)    onOK2=function(){  option="sum"  assign("option",option,envir=.GlobalEnv)  tkdestroy(win17)  inputs9()  }    ok_but2=ttkbutton(win17,text="OK",command=onOK2)    tkgrid(label_ok1)  tkgrid(ok_but2)  }    import_but=ttkbutton(win16,text="Select CSV file",command=getcsv)  ok_but=ttkbutton(win16,text="Next step",command=onOK)    tkgrid(label_import,sticky="w")  tkgrid(label1,sticky="w")  tkgrid(label2,sticky="w")  tkgrid(import_but)  tkgrid(ok_but)  }    opt1_but=ttkbutton(win15,text="Option 1",command=option1)  opt2_but=ttkbutton(win15,text="Option 2",command=option2)    tkgrid(label_options,sticky="w",row=0,column=0)  tkgrid(label_opt1,sticky="w",row=1,column=0)  tkgrid(label_opt2,sticky="w",row=2,column=0)  tkgrid(opt1_but,sticky="w",row=3,column=0)  tkgrid(opt2_but,sticky="w",row=3,column=1)    data_sample=data  column=ntotal+3  sample=data_sample[,4:column]  data_sample=cbind(data_sample[,1],data_sample[,2],sample)  colnames(data_sample)[1]="poly"  colnames(data_sample)[2]="gene"    i=3  for(i in 3:ntotal+2){  data_sample[,i]=as.numeric(data_sample[,i])  }  rm(i)  data_sample[,1]=as.character(data_sample[,1])  data_sample[,2]=as.character(data_sample[,2])  assign("data_sample",data_sample,envir=.GlobalEnv)  }  #  ###############################  # Window 18 #  # Parameter the rank products #  ###############################  #  inputs9=function(){  win18=tktoplevel()  tkwm.title(win18,"Rank product analysis: define the number of samples and replicates")    font=tkfont.create(family="Arial",size=12)  font_b=tkfont.create(family="Arial",size=12,weight="bold")  font_i=tkfont.create(family="Arial",size=12,slant="italic")    label1=tklabel(win18,text=paste("The ",nrow(data_pop)," genes will be ranked according to the enrichment in variant alleles"),font=font)  label2=tklabel(win18,text="\nAs the number of samples is too low, simulated groups (treated and control) will be generated",font=font_b)  label3=tklabel(win18,text="from the NGS results by several random samples with replacement",font=font)  label4=tklabel(win18,text="\nBy default, 1000 samplings will be performed",font=font)  label5=tklabel(win18,text="The value can be modified in the following window",font=font)  label6=tklabel(win18,text="\nThe variant frequency for each gene will then be compared with the general population",font=font)  label7=tklabel(win18,text="Treated vs general population and Control vs general population",font=font)  label8=tklabel(win18,text="\nODDS ratios will be determined",font=font_b)  label9=tklabel(win18,text="This step can be repeated several times",font=font)  label10=tklabel(win18,text="\nBy default, 4 replicates will be performed for both groups (treated and control)",font=font)  label11=tklabel(win18,text="The value can be modified in the following window",font=font)  label12=tklabel(win18,text= "\nFinally the ODDS ratios of the treated and the control group will be compared using the rank product method\n",font=font_b)    nsample=tclVar("1000")  nODDS=tclVar("4")    enter_nsample=tk2entry(win18,textvariable=nsample)  enter_nODDS=tk2entry(win18,textvariable=nODDS)    onOK=function(){  nsample=as.numeric(tclvalue(nsample))  nODDS=as.numeric(tclvalue(nODDS))  assign("nsample",nsample,envir=.GlobalEnv)  assign("nODDS",nODDS,envir=.GlobalEnv)  tkdestroy(win18)  inputs10()  }    ok_but=ttkbutton(win18,text="OK",command=onOK)    tkgrid(label1,sticky="w",row=0,column=0)  tkgrid(label2,sticky="w",row=1,column=0)  tkgrid(label3,sticky="w",row=2,column=0)  tkgrid(label4,sticky="w",row=3,column=0)  tkgrid(label5,sticky="w",row=4,column=0)  tkgrid(enter_nsample,sticky="w",row=4,column=1)  tkgrid(label6,sticky="w",row=5,column=0)  tkgrid(label7,sticky="w",row=6,column=0)  tkgrid(label8,sticky="w",row=7,column=0)  tkgrid(label9,sticky="w",row=8,column=0)  tkgrid(label10,sticky="w",row=9,column=0)  tkgrid(label11,sticky="w",row=10,column=0)  tkgrid(enter_nODDS,sticky="w",row=10,column=1)  tkgrid(label12,sticky="w",row=11,column=0)  tkgrid(ok_but)  }  #  ######################################  # No window, automatic calculations #  # Create the rank product functions #  ######################################  #  #1-With the mean of variant frequencies  #For the control samples  simcontrol_mean=function(dataset){  #Step 1: generate an artificial co-occurrence matrix based on the NGS results  #Create the initial dataset (only one sampling of "ncontrol" random samples)  end=ncontrol+2  #Create a vector of "ncontrol" numbers to select randomly some columns corresponding to the control samples  draw=sample(3:end,ncontrol,replace=TRUE)  #Select the "ncontrol" columns in the table data_sample (matrix) according to the vector "draw"  #A dataset with all the polymorphisms in rows and "ncontrol" columns with samples took randomly  #In this way, an artificial random distribution of the variants is obtained, depending on NGS results  dataset=data_sample[draw]  i=1  for(i in 1:ncontrol){  dataset[,i]=as.numeric(dataset[,i])  }  rm(i)  #Step 2: calculate variant allele frequencies for each polymorphism  #The sum of variant alleles for each polymorphism in the simulated control group is calculated  dataset$control_q=NA  i=1  k=1  for(i in seq(1,nrow(dataset))){  num=0  for(k in seq(1,ncontrol)){ #Columns corresponding to the control samples  if(dataset[i,k]==1){ #Identify the heterozygous individuals  num=num+1 #Add 1 to the sum variable for the next sample with a variant allele  }  if(dataset[i,k]==2){ #Identify the homozygous variant individuals  num=num+2 #Add 2 to the sum variable for the next sample with a variant allele  }  dataset[i,"control_q"]=num  }}  rm(i)  rm(k)  rm(num)  dataset$control_q=replace(dataset$control_q,is.na(dataset$control_q),0)  #The variant allele frequency is estimated  dataset$control_q=dataset$control_q/(ncontrol*2)  #A column with the polymophisms ID and the genes is added  dataset=cbind(data_sample[,1],data_sample[,2],dataset)  #Modify the first column names  colnames(dataset)[1]="poly"  colnames(dataset)[2]="gene"  #Step 3: calculate the mean of variant frequencies per gene  #Sum all the variant frequencies of all polymorphisms for each gene  dataset=ddply(dataset,.(gene),mutate,  control_q=sum(control_q))  #Create a column with the number of polymorphisms per gene  #Create the column "num" with "1" at each row  dataset$num=1  #Determine the number of polymorphisms per gene (sum of the column "num" per gene)  dataset=ddply(dataset,.(gene),mutate,  npolymorph=sum(num))  dataset$num=NULL  dataset$control_q=dataset$control_q/dataset$npolymorph  dataset$npolymorph=NULL  #As the values "control_q" are identical for each gene, replicates are suppressed. Only one row per gene is maintained  dataset$dup=duplicated(dataset$gene)  dataset=subset(dataset,dataset$dup=="FALSE")  #Add the frequency in the general population (p) to calculate the ODDS ratio (difference of variant distribution for each gene between both groups for each gene)  resultfinal=sqldf("SELECT distinct p.gene, p.p, r.control_q  FROM data_pop p  INNER JOIN dataset r  WHERE p.gene == r.gene  ")  #If the frequency for one gene in simulated control samples(control_q) is equal to 0, it is replaced with the frequency in the general population  resultfinal$control_q=ifelse(resultfinal$control_q==0,resultfinal$p,resultfinal$control_q)  #If it is equal to 1, it is replaced by 0.999  resultfinal$control_q=ifelse(resultfinal$control_q==1,0.999,resultfinal$control_q)  #The ODDS ratio is calculated for each gene: (control_q*1-p)/(p*1-control_q)  resultfinal$ODDS=(resultfinal$control_q*(1-resultfinal$p))/(resultfinal$p*(1-resultfinal$control_q))  resultfinal$control_q=NULL  rm(dataset)  rm(draw)  #The same step is reproduced "nsample-1" times  #By default, 1000 samples are performed  j=2  for (j in 2:nsample-1){  end=ncontrol+2  draw=sample(3:end,ncontrol,replace=TRUE)  dataset=data_sample[draw]  i=1  for(i in 1:ncontrol){  dataset[,i]=as.numeric(dataset[,i])  }  rm(i)  dataset$control_q=NA  i=1  k=1  for(i in seq(1,nrow(dataset))){  num=0  for(k in seq(1,ncontrol)){  if(dataset[i,k]==1){  num=num+1  }  if(dataset[i,k]==2){  num=num+2  }  dataset[i,"control_q"]=num  }}  rm(i)  rm(k)  rm(num)  dataset$control_q=replace(dataset$control_q,is.na(dataset$control_q),0)  dataset$control_q=dataset$control_q/(ncontrol*2)  dataset=cbind(data_sample[,1],data_sample[,2],dataset)  colnames(dataset)[1]="poly"  colnames(dataset)[2]="gene"  dataset=ddply(dataset,.(gene),mutate,  control_q=sum(control_q))  dataset$num=1  dataset=ddply(dataset,.(gene),mutate,  npolymorph=sum(num))  dataset$num=NULL  dataset$control_q=dataset$control_q/dataset$npolymorph  dataset$dup=duplicated(dataset$gene)  dataset=subset(dataset,dataset$dup=="FALSE")  result=sqldf("SELECT distinct p.gene, p.p, r.control_q  FROM data_pop p  INNER JOIN dataset r  WHERE p.gene == r.gene  ")  result$control_q=ifelse(result$control_q==0,result$p,result$control_q)  result$control_q=ifelse(result$control_q==1,0.999,result$control_q)  result$ODDS=(result$control_q*(1-result$p))/(result$p*(1-result$control_q))  result$control_q=NULL  result$gene=NULL  result$p=NULL  resultfinal=cbind(resultfinal,result)  rm(dataset)  rm(draw)  }    resultfinal$mean=apply(resultfinal[2:nsample+1],1,mean)  keep=c("gene","mean")  resultfinal=resultfinal[,(names(resultfinal)%in%keep)]  dataset_c=resultfinal    assign("resultfinal_c_mean",resultfinal,envir=.GlobalEnv)  assign("dataset_c",dataset_c,envir=.GlobalEnv)    rm(result,resultfinal)  }  #  #For the treated samples  simtreated_mean=function(dataset){  start=ncontrol+3  end=ncontrol+ntreated+2  draw=sample(start:end,ntreated,replace=TRUE)  dataset=data_sample[draw]  i=1  for(i in 1:ntreated){  dataset[,i]=as.numeric(dataset[,i])  }  rm(i)  dataset$treated_q=NA  i=1  k=1  for(i in seq(1,nrow(dataset))){  num=0  for(k in seq(1,ntreated)){  if(dataset[i,k]==1){  num=num+1  }  if(dataset[i,k]==2){  num=num+2  }  dataset[i,"treated_q"]=num  }}  rm(i)  rm(k)  rm(num)  dataset$treated_q=replace(dataset$treated_q,is.na(dataset$treated_q),0)  dataset$treated_q=dataset$treated_q/(ntreated*2)  dataset=cbind(data_sample[,1],data_sample[,2],dataset)  colnames(dataset)[1]="poly"  colnames(dataset)[2]="gene"  dataset=ddply(dataset,.(gene),mutate,  treated_q=sum(treated_q))  dataset$num=1  dataset=ddply(dataset,.(gene),mutate,  npolymorph=sum(num))  dataset$num=NULL  dataset$treated_q=dataset$treated_q/dataset$npolymorph  dataset$npolymorph=NULL  dataset$dup=duplicated(dataset$gene)  dataset=subset(dataset,dataset$dup=="FALSE")  resultfinal=sqldf("SELECT distinct p.gene, p.p, r.treated_q  FROM data_pop p  INNER JOIN dataset r  WHERE p.gene == r.gene  ")  resultfinal$treated_q=ifelse(resultfinal$treated_q==0,resultfinal$p,resultfinal$treated_q)  resultfinal$treated_q=ifelse(resultfinal$treated_q==1,0.999,resultfinal$treated_q)  resultfinal$ODDS=(resultfinal$treated_q*(1-resultfinal$p))/(resultfinal$p*(1-resultfinal$treated_q))  resultfinal$p=NULL  resultfinal$treated_q=NULL  rm(dataset)  rm(draw)  j=2  for (j in 2:nsample-1){  start=ncontrol+3  end=ncontrol+ntreated+2  draw=sample(start:end,ntreated,replace=TRUE)  dataset=data_sample[draw]  i=1  for(i in 1:ntreated){  dataset[,i]=as.numeric(dataset[,i])  }  rm(i)  dataset$treated_q=NA  i=1  k=1  for(i in seq(1,nrow(dataset))){  num=0  for(k in seq(1,ntreated)){  if(dataset[i,k]==1){  num=num+1  }  if(dataset[i,k]==2){  num=num+2  }  dataset[i,"treated_q"]=num  }}  rm(i)  rm(k)  rm(num)  dataset$treated_q=replace(dataset$treated_q,is.na(dataset$treated_q),0)  dataset$treated_q=dataset$treated_q/(ntreated*2)  dataset=cbind(data_sample[,1],data_sample[,2],dataset)  colnames(dataset)[1]="poly"  colnames(dataset)[2]="gene"  dataset=ddply(dataset,.(gene),mutate,  treated_q=sum(treated_q))  dataset$num=1  dataset=ddply(dataset,.(gene),mutate,  npolymorph=sum(num))  dataset$num=NULL  dataset$treated_q=dataset$treated_q/dataset$npolymorph  dataset$dup=duplicated(dataset$gene)  dataset=subset(dataset,dataset$dup=="FALSE")  result=sqldf("SELECT distinct p.gene, p.p, r.treated_q  FROM data_pop p  INNER JOIN dataset r  WHERE p.gene == r.gene  ")  result$treated_q=ifelse(result$treated_q==0,result$p,result$treated_q)  result$treated_q=ifelse(result$treated_q==1,0.999,result$treated_q)  result$ODDS=(result$treated_q*(1-result$p))/(result$p*(1-result$treated_q))  result$treated_q=NULL  result$gene=NULL  result$p=NULL  resultfinal=cbind(resultfinal,result)  rm(dataset)  rm(draw)  }    #Calculate the mean of ODDS ratios for the 1st replicate  resultfinal$mean=apply(resultfinal[2:nsample+1],1,mean)  keep=c("gene","mean")  resultfinal=resultfinal[,(names(resultfinal)%in%keep)]  dataset_t=resultfinal  assign("resultfinal_t_mean",resultfinal,envir=.GlobalEnv)  assign("dataset_t",dataset_t,envir=.GlobalEnv)    rm(result,resultfinal)  }  #  #2-With the sum of variant frequencies / size of the gene  #For the control samples  simcontrol_sum=function(dataset){  end=ncontrol+2  draw=sample(3:end,ncontrol,replace=TRUE)  dataset=data_sample[draw]  i=1  for(i in 1:ncontrol){  dataset[,i]=as.numeric(dataset[,i])  }  rm(i)  dataset$control_q=NA  i=1  k=1  for(i in seq(1,nrow(dataset))){  num=0  for(k in seq(1,ncontrol)){  if(dataset[i,k]==1){  num=num+1  }  if(dataset[i,k]==2){  num=num+2  }  dataset[i,"control_q"]=num  }}  rm(i)  rm(k)  rm(num)  dataset$control_q=replace(dataset$control_q,is.na(dataset$control_q),0)  dataset$control_q=dataset$control_q/(ncontrol*2)  dataset=cbind(data_sample[,1],data_sample[,2],dataset)  colnames(dataset)[1]="poly"  colnames(dataset)[2]="gene"  dataset=ddply(dataset,.(gene),mutate,  control_q=sum(control_q))  colnames(size)=c("gene","size")  dataset=merge(dataset,size,by="gene")  dataset$control_q=dataset$control_q/dataset$size  dataset$size=NULL  dataset$dup=duplicated(dataset$gene)  dataset=subset(dataset,dataset$dup=="FALSE")  resultfinal=sqldf("SELECT distinct p.gene, p.p, r.control_q  FROM data_pop p  INNER JOIN dataset r  WHERE p.gene == r.gene  ")  resultfinal$control_q=ifelse(resultfinal$control_q==0,resultfinal$p,resultfinal$control_q)  resultfinal$control_q=ifelse(resultfinal$control_q==1,0.999,resultfinal$control_q)  resultfinal$ODDS=(resultfinal$control_q*(1-resultfinal$p))/(resultfinal$p*(1-resultfinal$control_q))  resultfinal$p=NULL  resultfinal$control_q=NULL  rm(dataset)  rm(draw)  j=2  for (j in 2:nsample-1){  end=ncontrol+2  draw=sample(3:end,ncontrol,replace=TRUE)  dataset=data_sample[draw]  i=1  for(i in 1:ncontrol){  dataset[,i]=as.numeric(dataset[,i])  }  rm(i)  dataset$control_q=NA  i=1  k=1  for(i in seq(1,nrow(dataset))){  num=0  for(k in seq(1,ncontrol)){  if(dataset[i,k]==1){  num=num+1  }  if(dataset[i,k]==2){  num=num+2  }  dataset[i,"control_q"]=num  }}  rm(i)  rm(k)  rm(num)  dataset$control_q=replace(dataset$control_q,is.na(dataset$control_q),0)  dataset$control_q=dataset$control_q/(ncontrol*2)  dataset=cbind(data_sample[,1],data_sample[,2],dataset)  colnames(dataset)[1]="poly"  colnames(dataset)[2]="gene"  dataset=ddply(dataset,.(gene),mutate,  control_q=sum(control_q))  dataset=merge(dataset,size,by="gene")  dataset$control_q=dataset$control_q/dataset$size  dataset$size=NULL  dataset$dup=duplicated(dataset$gene)  dataset=subset(dataset,dataset$dup=="FALSE")  result=sqldf("SELECT distinct p.gene, p.p, r.control_q  FROM data_pop p  INNER JOIN dataset r  WHERE p.gene == r.gene  ")  result$control_q=ifelse(result$control_q==0,result$p,result$control_q)  result$control_q=ifelse(result$control_q==1,0.999,result$control_q)  result$ODDS=(result$control_q*(1-result$p))/(result$p*(1-result$control_q))  result$control_q=NULL  result$gene=NULL  result$p=NULL  resultfinal=cbind(resultfinal,result)  rm(dataset)  rm(draw)  }    resultfinal$mean=apply(resultfinal[2:nsample+1],1,mean)  keep=c("gene","mean")  resultfinal=resultfinal[,(names(resultfinal)%in%keep)]  assign("resultfinal_c_sum",resultfinal,envir=.GlobalEnv)  rm(result,resultfinal)  }  #  #For the treated samples  simtreated_sum=function(dataset){  start=ncontrol+3  end=ncontrol+ntreated+2  draw=sample(start:end,ntreated,replace=TRUE)  dataset=data_sample[draw]  i=1  for(i in 1:ntreated){  dataset[,i]=as.numeric(dataset[,i])  }  rm(i)  dataset$treated_q=NA  i=1  k=1  for(i in seq(1,nrow(dataset))){  num=0  for(k in seq(1,ntreated)){  if(dataset[i,k]==1){  num=num+1  }  if(dataset[i,k]==2){  num=num+2  }  dataset[i,"treated_q"]=num  }}  rm(i)  rm(k)  rm(num)  dataset$treated_q=replace(dataset$treated_q,is.na(dataset$treated_q),0)  dataset$treated_q=dataset$treated_q/(ntreated*2)  dataset=cbind(data_sample[,1],data_sample[,2],dataset)  colnames(dataset)[1]="poly"  colnames(dataset)[2]="gene"  dataset=ddply(dataset,.(gene),mutate,  treated_q=sum(treated_q))  colnames(size)=c("gene","size")  dataset=merge(dataset,size,by="gene")  dataset$treated_q=dataset$treated_q/dataset$size  dataset$size=NULL  dataset$dup=duplicated(dataset$gene)  dataset=subset(dataset,dataset$dup=="FALSE")  resultfinal=sqldf("SELECT distinct p.gene, p.p, r.treated_q  FROM data_pop p  INNER JOIN dataset r  WHERE p.gene == r.gene  ")  resultfinal$treated_q=ifelse(resultfinal$treated_q==0,resultfinal$p,resultfinal$treated_q)  resultfinal$treated_q=ifelse(resultfinal$treated_q==1,0.999,resultfinal$treated_q)  resultfinal$ODDS=(resultfinal$treated_q*(1-resultfinal$p))/(resultfinal$p*(1-resultfinal$treated_q))  resultfinal$p=NULL  resultfinal$treated_q=NULL  rm(dataset)  rm(draw)  j=2  for (j in 2:nsample-1){  start=ncontrol+3  end=ncontrol+ntreated+2  draw=sample(start:end,ntreated,replace=TRUE)  dataset=data_sample[draw]  i=1  for(i in 1:ntreated){  dataset[,i]=as.numeric(dataset[,i])  }  rm(i)  dataset$treated_q=NA  i=1  k=1  for(i in seq(1,nrow(dataset))){  num=0  for(k in seq(1,ntreated)){  if(dataset[i,k]==1){  num=num+1  }  if(dataset[i,k]==2){  num=num+2  }  dataset[i,"treated_q"]=num  }}  rm(i)  rm(k)  rm(num)  dataset$treated_q=replace(dataset$treated_q,is.na(dataset$treated_q),0)  dataset$treated_q=dataset$treated_q/(ntreated*2)  dataset=cbind(data_sample[,1],data_sample[,2],dataset)  colnames(dataset)[1]="poly"  colnames(dataset)[2]="gene"  dataset=ddply(dataset,.(gene),mutate,  treated_q=sum(treated_q))  dataset=merge(dataset,size,by="gene")  dataset$treated_q=dataset$treated_q/dataset$size  dataset$size=NULL  dataset$dup=duplicated(dataset$gene)  dataset=subset(dataset,dataset$dup=="FALSE")  result=sqldf("SELECT distinct p.gene, p.p, r.treated_q  FROM data_pop p  INNER JOIN dataset r  WHERE p.gene == r.gene  ")  result$treated_q=ifelse(result$treated_q==0,result$p,result$treated_q)  result$treated_q=ifelse(result$treated_q==1,0.999,result$treated_q)  result$ODDS=(result$treated_q*(1-result$p))/(result$p*(1-result$treated_q))  result$treated_q=NULL  result$gene=NULL  result$p=NULL  resultfinal=cbind(resultfinal,result)  rm(dataset)  rm(draw)  }    #Calculate the mean of ODDS ratios for the 1st replicate  resultfinal$mean=apply(resultfinal[2:nsample+1],1,mean)  keep=c("gene","mean")  resultfinal=resultfinal[,(names(resultfinal)%in%keep)]    assign("resultfinal_t_sum",resultfinal,envir=.GlobalEnv)    rm(result,resultfinal)  }  #  #################################  # Windows 19 and 20 #  # Generate the simulated groups #  # Calculate the ODDS ratios #  #################################  #  inputs10=function(){  win19=tktoplevel()  tkwm.title(win19,"")    font=tkfont.create(family="Arial",size=12)  font_b=tkfont.create(family="Arial",size=12,weight="bold")  font_i=tkfont.create(family="Arial",size=12,slant="italic")    label=tklabel(win19,text="The sampling and ODDS ratio calculations can take a little while\nplease wait for the window indicating the end of the calculations",font=font)    onOK=function(){  tkdestroy(win19)  #If the mean of polymorphism variant frequencies for each gene has been performed  if(option=="mean"){  #Random sampling for control samples "nODDS"*"nsample" replicates  simcontrol_mean()  control=resultfinal_c_mean  i=2  for(i in seq(2:nODDS)){  simcontrol_mean()  data=resultfinal_c_mean  control=cbind(control,data[,2])  }  #Random sampling for treated samples "nODDS"*"nsample" replicates  simtreated_mean()  treated=resultfinal_t_mean[,2]  i=2  for(i in seq(2:nODDS)){  simtreated_mean()  data=resultfinal_t_mean  treated=cbind(treated,data[,2])  }  final=cbind(control,treated)  assign("final",final,envir=.GlobalEnv)  assign("final_mean",final,envir=.GlobalEnv)  }  #If the sum of polymorphism variant frequencies/gene size has been performed  if(option=="sum"){  #Random sampling for control samples "nODDS"*"nsample" replicates  simcontrol_sum()  control=resultfinal_c_sum  i=2  for(i in seq(2:nODDS)){  simcontrol_sum()  data=resultfinal_c_sum  control=cbind(control,data[,2])  }  #Random sampling for treated samples "nODDS"*"nsample" replicates  simtreated_sum()  treated=resultfinal_t_sum[,2]  i=2  for(i in seq(2:nODDS)){  simtreated_sum()  data=resultfinal_t_sum  treated=cbind(treated,data[,2])  }  final=cbind(control,treated)  assign("final",final,envir=.GlobalEnv)  assign("final_sum",final,envir=.GlobalEnv)  }  win20=tktoplevel()  tkwm.title(win20,"")  label_end=tklabel(win20,text="The ODDS ratios were calculated, the rank product analysis can pe performed")    onOK=function(){  tkdestroy(win20)  inputs11()  }    ok_but=ttkbutton(win20,text="OK",command=onOK)    tkgrid(label_end)  tkgrid(ok_but)  }    ok_but=ttkbutton(win19,text="OK",command=onOK)    tkgrid(label)  tkgrid(ok_but)  }  #  ######################################  # Windows 21 and 22 #  # Perform the rank product analysis #  ######################################  #  inputs11=function(){  win21=tktoplevel()  tkwm.title(win21,"Rank product analysis: results")    font=tkfont.create(family="Arial",size=12)  font_b=tkfont.create(family="Arial",size=12,weight="bold")  font_i=tkfont.create(family="Arial",size=12,slant="italic")    label_tables=tklabel(win21,text="Two tables are obtained.\nThe first one indicates the genes enriched in treated samples\nThe second one indicates the genes enriched in control samples\n",font=font_b)  label_options=tklabel(win21,text="Two options",font=font)  label_ngene=tklabel(win21,text="Set the number of top genes ranked in the tables (default: 10)",font=font)  label_cutoff=tklabel(win21,text="Set a cutoff value (default: pfp=0.05)\n",font=font)  label_graph=tklabel(win21,text="Graphs can also be generated",font=font_b)  label_cutgraph=tklabel(win21,text="Set a cutoff value (default: pfp=0.05)",font=font)  label_parameters=tklabel(win21,text="\nParameters can be modified in this window to generate new tables/graphs\nEnter 'Submit' to validate the parameters and generate the tables/graph\n",font=font)  label_results=tklabel(win21,text="Results can be visualized in R workspace\nEnter 'topgenes' to see the tables and 'graph' to see the graphics",font=font)  ngene=tclVar("10")  cutoff=tclVar("0.05")  cutoff_graph=tclVar("0.05")    enter_ngene=tk2entry(win21,textvariable=ngene)  enter_cutoff=tk2entry(win21,textvariable=cutoff)  enter_cutoffgraph=tk2entry(win21,textvariable=cutoff_graph)    #Two class analysis: control vs treated, detect the difference of enrichment in variant alleles  gene=final[,1]  final[,1]=NULL  i=2  test.cl=0  for(i in seq(2:nODDS)){  test.cl=c(test.cl,0)  }  rm(i)  i=1  for(i in seq(1:nODDS)){  test.cl=c(test.cl,1)  }  rm(i)  RP.out=RP(final,test.cl,rand=123,plot=FALSE)    submit_gene=function(){  ngene=as.numeric(tclvalue(ngene))  topgenes=topGene(RP.out,num.gene=ngene,gene.names=gene)  assign("topgenes",topgenes,envir=.GlobalEnv)  win22=tktoplevel()  label_ok=tklabel(win22,text="The tables have been generated\nEnter 'topgenes' in R workspace",font=font)  OK_but=ttkbutton(win22,text="OK",command=function()tkdestroy(win22))  tkgrid(label_ok)  tkgrid(OK_but)  }    submit_cutoff=function(){  cutoff=as.numeric(tclvalue(cutoff))  topgenes=topGene(RP.out,cutoff=cutoff,gene.names=gene)  assign("topgenes",topgenes,envir=.GlobalEnv)  win22=tktoplevel()  label_ok=tklabel(win22,text="The tables have been generated\nEnter 'topgenes' in R workspace",font=font)  OK_but=ttkbutton(win22,text="OK",command=function()tkdestroy(win22))  tkgrid(label_ok)  tkgrid(OK_but)  }    submit_graph=function(){  cutoff_graph=as.numeric(tclvalue(cutoff_graph))  par("mar")  par(mar=c(2,2,2,2))  graph=plotRP(RP.out,cutoff=cutoff_graph)  assign("graph",graph,envir=.GlobalEnv)  win22=tktoplevel()  label_ok=tklabel(win22,text="The graph has been generated\nEnter 'graph' in R workspace",font=font)  OK_but=ttkbutton(win22,text="OK",command=function()tkdestroy(win22))  tkgrid(label_ok)  tkgrid(OK_but)  }    end=function(){  tkdestroy(win21)  }    submit1=ttkbutton(win21,text="Submit",command=submit_gene)  submit2=ttkbutton(win21,text="Submit",command=submit_cutoff)  submit3=ttkbutton(win21,text="Submit",command=submit_graph)  end_but=ttkbutton(win21,text="End",command=end)    tkgrid(label_tables,sticky="w",row=0,columnspan=3)    tkgrid(label_options,sticky="w",row=1,column=0)  tkgrid(label_ngene,sticky="w",row=2,column=0)  tkgrid(enter_ngene,sticky="w",row=2,column=1)  tkgrid(submit1,sticky="w",row=2,column=2)    tkgrid(label_cutoff,sticky="w",row=3,column=0)  tkgrid(enter_cutoff,sticky="w",row=3,column=1)  tkgrid(submit2,sticky="w",row=3,column=2)    tkgrid(label_graph,sticky="w",row=4,column=0)  tkgrid(label_cutgraph,sticky="w",row=5,column=0)  tkgrid(enter_cutoffgraph,sticky="w",row=5,column=1)  tkgrid(submit3,sticky="w",row=5,column=2)    tkgrid(label_parameters,sticky="w",row=6,columnspan=3)  tkgrid(label_results,sticky="w",row=7,columnspan=3)  tkgrid(end_but)  }  #  inputs1() |
| --- |
